# Appendix

**Published:** 1838-04-01

**Authors:** 


### APPENDIX.

### MR. GUTHRIE'S CLINICAL LECTURES.

### WESTMINSTER HOSPITAL.

### On Compound and Gun-shot Fractures of the Arm.

*Delivered December 30, 1837.*

The man I now place before you is one of the heroes of Irun, at which place he received a musket-ball, which broke the right arm nearly mid-way between the shoulder and the elbow. It does great credit to the medical officers engaged on that service, and particularly to our friend Alcock, (who was educated at this hospital,) and proves what a man can do who is attentive to his studies and his duties. The arm is a little bent outwards, which has arisen from that army not having been supplied with proper splints; and it is not sound, from there being still some dead bone to come away. I feel it readily with the probe for the space of three inches or more. You shall see me remove this.

It is done, gentlemen, more quickly than I can relate it. I made an incision, four inches long, in the line of the ulceration, down to the bone on the inner edge of the insertion of the deltoid, and downwards on the outer edge of the biceps. By this cut I gained but an incision,—a fact you must never forget, as it shews the difference between parts in their natural and diseased state. In order to expose the bone, the thickened, hardened parts, almost resembling cartilage, were separated from it with the knife and finger, which being done, I easily removed two pieces of dead bone, worm-eaten in appearance, indicating the action of the absorbents on them. There does not seem to be any more, and we must therefore hope he will soon get quite well, and save an arm, and have it equal to useful labor, which would probably have been cut off by many surgeons thirty years ago. Let us inquire into these cases more fully.

A compound fracture is, as you all know, a broken bone which protrudes, or has protruded, through the skin, causing thereby a rent in the integuments, which, when the broken bone is brought back to its proper place, may perhaps unite, if the cut edges are placed in apposition. If it should do so the compound fracture becomes a simple one, and the cure is effected by a very easy, natural process. If it should not unite, the wound remains open, and the process of cure is much more complicated. A gun-shot fracture must always be a compound fracture, because the shot-hole cannot unite or heal by the adhesive inflammatory process: but a compound fracture caused by a heavy cart-wheel going over the part must be a more dangerous injury than any musket-ball fracture. The treatment therefore in all serious cases, is pretty much the same.

In a gun-shot fracture, the bone or bones may be more or less broken, the splinters larger or smaller, of a greater or less extent. Let us now, however, confine ourselves to the upper arm, and suppose that the ball has done the least possible mischief; it has gone through, or remains, having merely broken the bone transversely. In the first place examine the wound at the moment, if you can, and in the gentlest manner. The weight of the fore-arm usually keeps the lower end of the bone tolerably well in its place, and you ascertain the evil by gently feeling the part all round. Your finger should then be passed into the shot-hole, and if it will not go in, the opening must be enlarged, so as to enable it to enter freely. The object is to ascertain the state of the fracture, and to remove the splinters, and the extent of the incision must depend on them. This is the true principle of what has been called dilatation in wounds; for to cut a man because he has been shot is absurd; he should only be cut for some good and specific reason. If, for instance, the ball has merely struck the bone, and has passed out, causing a transverse fracture only, there is no necessity for dilating such a wound at the moment, although it must be done at a later period; it is time enough to do it when necessary, and it is better done then; for if done at first, the cut would have closed up before any bone would be ready to come away. This is one of the differences between a compound fracture from a slight accident, and a gun-shot fracture. In the accidental fracture, the force being often applied to the ends of the bone, no injury is done to the broken part by direct collision. It is always done in the simplest gun-shot fracture: the ball grazes and injures the bone, depriving it of a part of its periosteum, and a scale or portion will be separated at that place; but this is a process requiring time, and it is to allow of the free passage of this exfoliated bone that an incision must be made when necessary. If the ball lodges in the soft parts after breaking the bone, an incision or incisions are required for its removal; and an incision must also be made if you should fear the lodgment of matter, a free vent for which is always of the greatest importance. Receive it as a general rule, that whatever may require to be done the first few days, had better be done on the first than on the second, for after inflammation has commenced, any handling or examination, however gently made, gives much greater pain. Suppose, if you please, the bone to be greatly splintered; an incision sufficiently large and deep to enable you to remove the splinters will be necessary, and they should all be removed if it can be conveniently done. Add to this a wound of the brachial artery, on which you must place two ligatures, one above the wound, and the other below it. You will now perhaps be able to put your fingers quite through the arm, and the wound will look very ugly; but you may yet add a shot through the fore arm, you may even break a bone of it, or knock off a thumb or a finger or two; nevertheless the arm must not come off: it is only sixty seconds or so between an arm on, and an arm off; but as a man is not a crab, and cannot reproduce a limb, he must run great risks to keep it.

The peculiar danger from a gun-shot fracture, and which is not so great in a common compound fracture, arises from inflammation taking place in the membrane lining the shaft and the cancellated structure of the bone. When this inflammation exceeds a certain point, it causes the death of the bone which it lines and nourishes, and gives rise to a corresponding, but peculiar and different action in the membrane or periosteum covering and nourishing the bone externally. This swells, thickens, and begins to deposit new bony matter, not only in its own structure, but external to it, and induces the neighbouring vessels to take on a similar action, to the extent often of an inch or more. This ossific deposition begins early. I have seen it by the 20th day, and it begins earlier, and by the end of the fourth week it is often very remarkable. When our own sick and wounded were in due order, after the battle of Toulouse, in the old convents of the town, I thought it right to make the acquaintance of the physicians and surgeons of the Civil Hospital. They received me and my principal friends with the greatest kindness, and invited us to witness several of their operations for different complaints. On my inviting them, in return, to see our surgeons amputate three thighs, which was all they had to shew, the gentlemen I requested to do them, came to me in the most mutinously complimentary manner, to beg I would operate myself, to which, as I found they were determined not to do it, and as their intentions were good, I was obliged to accede. Every leg was off in less than 80 seconds; but tying 10 or 12 arteries was a more troublesome matter. They were all in the fourth week after the injury; and each vessel nearer than an inch and a half to the bone was so surrounded by ossific matter, deposited by the action I have alluded to, that the ligatures on being drawn tight, sounded as if cutting a chalky substance, and did even in some instances cut through. The two preparations of the arm bone I now shew you, once belonged to two gallant soldiers of Waterloo. Mr. Morel cut off one, I amputated the other at York Hospital in 1816. They are each twice as thick and as clumsy as an ordinary bone, perforated in several places by holes, and containing in many parts pieces of the original bone. The new surrounding case of bone is hollow, its wall is in some places half an inch thick, of a deep brownish yellow color; the old original bone is white, thin, and as if worm-eaten. It is a case of necrosis, with its sequestra, you will say; but mark the difference. The five inches of bone I now shew you, I took out of a young man's thigh last year in this hospital; the new formed bone surrounding it being more than half an inch thick. After making a hole in it with the trephine, I was obliged to break it down with a mallet and chisel, used even with great force, before I could withdraw this dead piece. The dead bone or sequestrum is in these cases of natural necrosis in young persons, in one piece, even if the whole shaft is implicated, and may in general be readily drawn out when sawn through the middle by the trephine. The young man got well.

In a gun-shot fracture, as you see in these preparations, the old bone is in several pieces, and could only have been drawn out after much chiselling at several places; and the articulating surface of the humerus in the joint is diseased, I presume from the extension of inflammation. The danger you have to dread is the shutting up of these splinters of bone by the newly-formed ossific matter; and the object you ought to have in view is the prevention of the extension, if not the establishment of this, which is to a certain extent a necessary and consolidating process. This is to be done by the suppression of the inflammation in the internal part of the shaft of the bone particularly, and of the arm generally. The splinters should be removed if they will admit of it; the arm should be placed at perfect rest if possible in the bent position, duly supported by proper splints, or otherwise as the case may require. Vascular action must be subdued, by the application of cold and by leeches, until cold seems to be disagreeable, when warmth is to be substituted. When suppuration is fully established, the remaining splinters may be very gently sought for and removed; but if this cannot be done, and time runs on, the firm thickening of the arm begins to indicate the deposition of new formed bony matter; and after a further lapse of time, a particular spot of inflammation at some part implies the formation of an abscess in the new bony deposit. It is caused by the irritation of a dead splinter; and when the abscess breaks externally, the probe will pass through it, and through such a small hole, as you see several in these two preparations, and rests on the rough dead splinter. If you have not found this out before, you have found it now, and the dead bone must be removed. The earlier it is done, the softer the deposit, which in such cases will cut like Parmesan cheese intermingled with lime. Such was the practice of those in the Peninsula, who knew what they were about. The great difficulty I had to encounter was to teach, or rather to unteach, the young gentlemen who came to us from home. Those that knew any thing were mostly filled with theory not founded on practical information; and many of their masters, when they condescended to notice us at all, have, when they learned a little better themselves, attributed the mal-practices of their own students to us. You may perhaps think, from what you have heard and read, that the case I have just shewn you from Roliça is an accidental one. If you will inquire for Colonel Hodge, in the Spring, at the United Service Club, he will readily shew you his arm, broken by gun-shot on the same day. It is even a better one.

During the retreat from Talavera, the wounded who could walk over the bridge of Arzobispo were afterwards collected at the convent of Deleytosa for treatment. Many underwent operations, and many lost their lives for want of proper care and conveyance during this short march of half a dozen days. The broken arms were mostly amputated. I objected to some few undergoing this operation, and saved them for that day; but I lost a friend thereby who never cordially forgave me even unto his death.

At the battle of Albuhera, Sir Gregory Way, now a major-general, was shot in the left arm, which was broken in the upper third at that place which Baron Larrey and the French surgeons of the day say, rendered amputation necessary. I desired that it might not be done. I have not examined it since it left my care on moving from the village of Valverde, and I believe the shoulder is stiff, but the use of the under part of the arm seems perfect. The last instance I shall give you is that of the present Master-General of the Ordnance, Sir Hussey Vivian. He was also shot in the left arm, and had it broken before Toulouse. Sir James M'Grigor was with the head-quarters, on the left bank of the Garonne, and was pleased to depute me to act as chief on the right, where the principal part of the contest lay. The officers under whose charge he immediately came, (and they were well taught men at home,) decided for amputation; but knowing I was on the field, they deferred for my opinion. On moving into Toulouse, after the battle, he had the able assistance of Mr. Gunning, and regained as good an arm as he had before.

I shall finish this part of my lecture with the following very general conclusions.

1. An upper extremity should not be amputated for almost any accident or accidents that can reasonably happen to it from musket-shot. Whenever it is done a special report should be called for by the Inspector-General of Hospitals, and the broken bones and other cause for amputation should accompany it.

2. If the head of the arm-bone entering into the composition of the shoulder-joint is broken to pieces, that portion of the bone should be sawn off, but the arm must remain. The patient will have all the under use of it.

3. If the elbow-joint is shot through, it is to be cut out, and the fore-arm brought into the bent position. The sufferer will have a very good and useful arm.

4. A fore-arm will bear so much fracturing and cutting, at the time and afterwards, that it should not be amputated without a special report of the reasons, with the broken bones, being sent to the inspector.

5. A thumb and a finger, or two fingers, are worth saving: but a serious injury to the wrist-joint generally requires amputation.

The treatment of gun-shot fractures of the fore-arm must be conducted on the same principles as those of the upper arm. The fore-arm must be placed in the half-bent position, or at a right angle with the arm. The thumb must be uppermost, the hand not supported, and two solid splints wider than the arm, and duly padded, should be placed one on each side. The arteries when wounded must be secured at each end, according to the principles laid down in my work.* The danger arising from necrosis is to be carefully attended to, and the motions of supination, and pronation must, if possible, by early attention be preserved.

The splints used for the upper arm should be made of solid wood or tin, firm although light. The anterior one should be made at a right angle, which angle should correspond with the bend of the arm. The anterior piece, or that for the fore-arm, should be hollowed, so as to admit of a very slight degree of pronation, as it is painful and not necessary that the hand should be quite supine.

7. If the surgeon does not know the anatomy of the parts well, he had better cut off the arm.

---

* On the Diseases and Injuries of the Great Arteries of the Human Body, with the Operations required for their Cure. 1830.

I have pointed out to you the miserable and desolate state in which the medical officers, not attached to regiments, are situated in a campaign; from which you will perceive how necessary it is that some amelioration should take place in their condition; not so much on their own account, as on that of the persons committed to their care. These poor creatures suffer in a manner it is quite deplorable to think of, and which, I must say, is a disgrace to the character of the country, and to every man and woman in it possessing one spark of the common feelings of humanity. If an unhappy wretch of a doctor has to travel two-thirds of a day, generally on foot, at the tail of a cart of any kind, shivering and wet to the skin, without food, with scarcely a dry change of clothing, with no one to help him in the common necessaries of life, how can he attend to his sick and wounded? It is impossible. Self-preservation is the first law of nature. He must ascertain where he is to eat and sleep, how his clothes are to be dried, to seek food for himself and his beast, if he has one, and to do every thing, if he wishes to live himself, except attend to his professional duties. On his arrival at the halting-place, he ought to see his people housed, put to bed if possible, and give directions for their food, make up their medicines, see them administered, bleed them if necessary, or dress their wounds. A man worn with his day's labor, or however tired under a burning sun, may do all this for two or three hours more, if he has a hope of a tolerably comfortable place to rest himself in, or of something to eat, but not otherwise. At night he should again visit his sick, and arrange for a move shortly after daylight next morning, when all these duties are again to be repeated before the march is begun. Under existing disabilities, no man, with the enduring strength even of a jackass of Malta, or of Old Castille, could do it, and the sick must be neglected, and they have been neglected whenever the doctor was unequal to the duties.

Many a brave and gallant soldier lost their lives from the want of that attendance, and care I have alluded to. Many a desolate and unhappy mother has mourned the loss of a son she need not have mourned for; yet England is the greatest nation upon earth, she professes to be the most civilized, the most humane, and did give twenty millions sterling for the relief of the negroes. It is to be hoped, however, that the 658 gentlemen who hold the purse-strings of the country may learn the truth, and I have full confidence that when they do, they will draw them on this point liberally, so that its gallant defenders may on such occasions feel that its boasted humanity is not only for the ear.

There are two ways of proceeding in order to enable the junior medical officers of the army to do their duty to the sick and wounded committed to their charge. One is by giving them an allowance for a servant, under ordinary circumstances, whether at home or abroad, and by granting them a soldier-servant in addition, on service, in whom they may repose the confidence of not being materially ill-treated unless he deserts. I will hereafter shew where this servant may come from with advantage to all parties. The second mode of proceeding is by much the best, viz., of appointing three assistant-surgeons to each regiment on active service. A young man taken into the army at 21 years of age, which I hope will soon be the age at which he can obtain his diploma as a surgeon, knows nothing of the management of sick, or of providing for them in any way; nor of the character and tricks of the soldier, however good his classical and general attainments may be, in addition to his professional knowledge. He is called upon to regulate and command upon points which he does not understand, and therefore does not do, and he has a great part of his duty to learn when he ought to be putting it into practice. He is comparatively inefficient. The assistant-surgeon of a regiment learns the duty of a soldier in addition to that of a doctor, and a military surgeon ought to know one just as well as the other. I always found in Spain that three assistant-surgeons of regiments were worth four assistant staff-surgeons or mates who had not been attached to regiments. They know how to manage the soldier, as well as to physic him; they go into action with him as a point of honor, willingly and cheerfully; they attend him kindly and feelingly afterwards. They are equally under the control of the head of the medical department; he can and ought to be able to rule them as he pleases, and although he may often send them to the rear, their tendency is to return to their homes with the regiment in the front. The tendency of the hospital mate or assistant is, on the contrary, to get away to the rear, where he may perhaps find something like a little comfort. The expense of each man is the same.

I once ordered one of the best assistant-surgeons I had, to the rear with a large party of sick; when I was informed that his commanding officer would not allow him to take his servant with him, being one, although a bad way of inflicting a punishment which he perhaps had deserved. I remonstrated, saying it was my sick must suffer, and that I would have my duty done; but I did not succeed: and in order to shew my feeling on the subject, I sent another assistant-surgeon out of his regular tour of duty. The general of division would have settled the matter in a second, but I did not choose to do it in that way, for it would have made us bad friends on a point of duty not strictly medical; and I was on such good terms generally with all the commanding officers that they never disputed any thing I desired when strictly in my own province. You will hardly believe, that an hour after daylight, when it was quite plain the enemy were quiet in their quarters, I have desired one wing of a regiment to take two tartar emetic pills, and the other wing two calomel pills, and have seen it done, being the best proof I can give you of the confidence of the officers, and of the discipline of the troops. You must learn on service to prevent a fever, as well as cure it, or at all events to be prepared to meet it in the best possible manner. A short time after we had a small fight, and the commanding officer of the regiment in question, a very distinguished soldier, was wounded towards the evening; and as a commanding officer, when shot through the body, becomes nobody, he was pretty much at the mercy of the doctors, or rather at mine, for I took good care his own regimental officers should not stir from the regiment. He was not the worse off for being in my clutches, as I wished only to read him a gentle practical lesson, without almost making him feel it. I removed every body from the village except himself, knowing he had no conveyance, and allowed all the troops to pass except the rear. It was now late at night, and he sent to ask whether I really did mean to let him fall into the hands of the enemy. This was all I wanted, as I had always intended he should accompany me. I therefore went to him, saying I had come for him, as I had arranged; but he had no means of moving, so I offered to lend him my horse, by the side of which I walked until morning. We never alluded to the old affair; but we never had a difference about doctoring afterwards; and he has since paid me, in London, the highest compliment in his power, viz., he placed his life in my hands, under circumstances of great difficulty, and he escaped. If he should ever serve again I am sure he will cherish well his doctors, if they deserve it, and I care nothing for them if they do not.

I remember a village on the great plain of the Guadiana, near Merida, in which three regiments were quartered in the sickly season in the Autumn, when fever prevails. Three rows of hillocks marked the last resting-place of the dead on earth, and my attention was attracted by one row being much shorter than the other two. I found, on inquiry, that the regiments were very much of the same strength, and quite under the same circumstances. The doctors were equally able; two were men entering rather on the middle period of life, the third was a very young man, and perhaps the worst doctor of the three; but the short row of tumuli belonged to him. I was very desirous of making this out, and after carefully visiting all the hospitals and quarters, I ascertained the reason. He was the better soldier, if not the best doctor. His hospitals were in better order, the materiel was more perfect, the labour bestowed on every part, except in physic, was greater, and five per cent at least of human life was the saving and the result. I never saw it otherwise.

A staff-surgeon, who has not been a regimental officer, has this kind of duty to learn; and if he tries it a little late in life he rarely learns it, or at all events, rarely practises it, and from five to ten per cent. loss of human life is the consequence. It was the custom, at the commencement of the last war, to appoint gentlemen who had influence at home to the offices of staff-physician and surgeon, totally overlooking the merits of those who were serving, and had therefrom just claims for those appointments. This error has been corrected, but the situation of the staff-officer has not been improved as it ought to have been; he now knows his duty it is true, but has not the means of doing it as it ought to be done on service. He is gazetted a staff-surgeon, which is stated to be a promotion; and every soldier, on being promoted, knows he attains a step of rank even if it be only from corporal to serjeant; but as the medical officer can hold no direct military rank or command, a relative rank is given him, which is of use only as it regulates his quarters, his baggage, his pension for his widow, his prize money, his horses, &c., and a regimental surgeon has this rank as a captain. When promoted, it is but natural to think he would then gain something by such promotion, as all other soldiers do; but no, he is still to be a relative captain. He is to have no servant, but is allowed five shillings a week to find one, when he can catch either the money or the man. In the mean time, he is appointed to a duty which in the field he can hardly do well, without four animals, and certainly with not less than three, and two servants, a proportion allowed to every staff-officer of the military branch. The staff-surgeon should always be promoted or made on account of his knowledge of anatomy, medicine, and surgery; he ought to be the ablest of the officers of the corps, and imbued with the zeal and spirit of a soldier in all that concerns them, and his department. He should therefore be promoted for his merit, and should feel that he has gained something, at least in rank, and privileges, as his reward. So far from this being the case, he will find his expenses increased, his means rather diminished, his comforts greatly reduced. After years of service, any regimental surgeon can exchange with him on equal terms, and is equally eligible for promotion; an arrangement which has been made of late years for the sake of economy. It is not then wonderful that few regimental surgeons can be found who will take a staff-surgeoncy, unless to escape the West Indies for a time; and that few cavalry surgeons will even take a deputy-inspectorship of hospitals, unless they can be employed as such. The office of staff-surgeon is therefore one nobody wishes to have, unless for some particular purpose of his own, unconnected with the good of the service; and then those gentlemen are generally too old or too idle for any severe, and active duty. When this occurs they would rather return to a regiment, and thus the promotion of the juniors is arrested. The last assistant-surgeon promoted to a surgeoncy with whom I am acquainted was 26 years an assistant, the one a little before him was 28 years an assistant, and it is not very uncharitable to suppose, that at 50 odd years of age he may be unequal to the duties he may have to perform. I have no hesitation in saying, that any such person will be unequal to the duties of a regimental surgeon before the enemy, if continued for any length of time. I shall not enlarge on this subject, but merely remark to you that the medical and surgical duties of the Peninsular war were done, when they were well done, by men from 20 to 35 years of age. An elderly man must be an indifferent operating surgeon unless he is in the constant practice of his profession as such; and even then he cannot bend his back as long and as often as is required; he is like an elderly captain or major of infantry, disposed to get a cough, the rheumatism, or the gout, to say nothing of a stomach-ache after a night or two's exposure to a heavy rain, and to find his way to drier lodgings in the rear. If middleaged men could be found of the iron mind and frame of my excellent and kind friend Lord Lynedoch, my observations would be worth nothing, but I am afraid there are, and have been few in the world possessing his ardour and principles.

Of the senior branches of the medical department, I shall only say they are worse treated than the juniors, but my old and able friend, Sir James M'Grigor, understands this subject better than I do, and will I hope be able to improve their situation. There is no man has more love for his profession, more kindness of heart, a greater desire to act fairly and honorably to every one, but he is without the power of granting promotion except to very few. With assistant-surgeons of 28 years standing, and every other situation in proportion, a man must be almost an angel to please anybody, and more than an arch-angel to please everybody. His office is one that nobody can envy him under such circumstances.

Lest it should be supposed that, in the foregoing observations on army doctors, I should mean indirectly and covertly to censure any particular persons, which is not my object, I shall proceed to shew you how the Legislature has thought fit to treat the civil doctors, and I do not think you will find much difference.

When a convicted felon is found previously to execution to stand in need of surgical assistance, it is provided by Act of Parliament that he shall be attended by a man possessing the only known qualification in the empire in surgery, viz. a diploma from one of the three Royal Colleges of Surgeons. Before a gentleman can be an assistant-surgeon in the army he must have the same qualification; and it is also required from a surgeon in the navy. By the Poor Law Bill this qualification, small as it is, is laid aside for the poor, thus placing them in a worse situation than a convicted felon, or a soldier, or a sailor. It is stated instead of that qualification, that the doctor to attend them shall be a medical man duly licensed to practise, for which person the Legislature must send to Australia, from whence he may come perhaps with the black swans, there being no such man known in Great Britain or Ireland. In these countries a man may be either physician by a degree, surgeon by diploma, or apothecary by license; but no one qualification embraces the others, and the words medical man are just as indefinite as they would be found in the law, if the word lawyer were used to describe that class of men as fitted for all the higher offices. The misfortune however is, that it has pleased the Executive to understand, that the Legislature meant by the words, medical man, an apothecary, because he has a license to practise as an apothecary, and the Boards of Guardians, acting under the Act of Parliament, take an apothecary, and make him a surgeon by their own authority, and agree with him to do the higher operations of surgery, which he is not qualified to perform by any other known authority, at the lowest price they can grind him down to; thus placing the poor man when sick, in a worse situation than a convicted felon. By this reading of the Act of Parliament, gentlemen, oftentimes illiterate, are enabled to allow the most ignorant to compete with the most able, and to make the decision depend not on ability, but on the saving of money. An Act worthy of the lowest state of barbarism in any country, and very disgraceful to the Parliament which enacted such a law. The only excuse for them is, that they did not know what they were about; that they took it for granted, the designation of medical man was good, and sufficient. If it had been a matter connected with the Church or the Law, the heads or authorities in these professions would have been consulted by the person at least who drew the Bill; but being a matter only of human life as connected with good medical treatment, every gentleman thinks himself entitled to dabble in it. The chief commissioner, Mr. Frankland Lewis, who possesses one of the best and kindest hearts in the world, has done his best to remedy the evil, but he cannot give a new reading to the law. There are also many places in which persons qualified to act as surgeons or even apothecaries cannot be found, and no one can desire impossibilities; but even this is the fault of the Legislature. Five years have elapsed since a Bill for the Regulation of Surgeons and Apothecaries in England, and Wales, was brought into the House of Commons, by the authorities of these two bodies, who agreed in its provisions, with one addition, to be afterwards made in Committee. This would have rendered the public great service, and removed various disabilities, and vexations under which the profession labours, and have prevented many of the difficulties which have since taken place, and its defects when clearly shewn might have been amended. It was opposed by Mr. Warburton, and others, and turned over on the plea, that a more extensive enquiry was necessary before a good, and comprehensive Bill could be framed, capable of removing every grievance. What followed? Mr. Warburton had his Committee the year after; it sat, or rather he sat nearly alone, as chairman, and Committee for several months; he published three large volumes of evidence, but has made no report; and for five years therefore the public has been deprived of the advantages which would have been obtained from the rejected Bill. This is not all. The College of Surgeons of London, instead of advancing and improving with the times, has stood still, waiting for Mr. Warburton's measures. Instead of increasing its demands on the candidates for its diploma, as had been done every two or three years for the last fifteen years, and as it would have done long since; instead of insisting on a short but sufficient bonâ fide education, in the place of a long, idle, and insufficient one, by which both parents, and the public would have been greatly benefited, they have done nothing. That they have done so is not my fault. Mr. Warburton cannot make a report from his evidence, which shall be founded on fact, because a part of that evidence is entirely hearsay matter; many parts which refer to facts are not true for the same reason, and on one side only was there any cross-examination. A Committee of the House of Commons, desirous of knowing the truth, and being totally unacquainted by education with the subject, ought to have proceeded like an election-committee, and have allowed each party implicated, whether appellants, or defendants, to have had one such medical counsel as they pleased, with fair right of cross-examination in the usual manner. The committee might then perhaps have made out the truth, but as people wickedly say that small evidence will sometimes enable even an election-committee to decide a question, I fear a committee of the House of Commons, as it is at present constituted, is not the best mode of proceeding, in order to elicit it in medicine.

I shall conclude this subject by drawing your attention to the fact, that neither the lawyers, nor the churchmen of the three kingdoms, have been able to form one code of law, or of religion, for them. Nor can any one form one code in medicine, without repealing all the Acts of Parliament, and abrogating all the Charters which have been granted by various kings for three or four centuries past; a proceeding which I do not think the Government will recommend, or the Legislature sanction. You will perhaps ask me what can be done under such circumstances? the answer is simple, and the mode of proceeding as much so. Let a secretary of state, or a chancellor of the exchequer, write to the different Universities, Colleges of Surgeons, and Societies of Apothecaries, desiring them to communicate with each other, and with him, on such points as they would wish to have settled by Act of Parliament, for their mutual advantage and that of the public. These Bodies would, I suspect, soon come to a right understanding, as far as their own interests were concerned, and I hope those of the public. The Colleges of Surgeons of London, Dublin, and Edinburgh, feel their respective disabilities too strongly, not to embrace readily any proposition which would lead to their final settlement. The other two bodies would not, I think, be very intractable. The opponents on all sides should even have fair play, and any great functionary would be able to settle the matter, both civil and military, to the advantage of all parties and of the public, with very little trouble, in less than two months. But no functionary, whether Whig or Tory, can find time to trouble himself about it; and the public must continue to suffer, until some one shall be found who may have time, and humanity enough to undertake it.

The campaigns of 1793 & 4, followed by that of the Helder in 1799, seemed to have left little impression on the minds of the medical chiefs of the army in 1808, as to the wants of the sick, and wounded soldier; and very little more on the minds of those whose duty it was to attend to the conveyance of troops to the scene of action at the commencement of the war. The troops were embarked in transports, maintained at a great expense, and commanded by men who were oftentimes quite incompetent to the charge committed to their care; and the loss of ships, and the loss of lives, which frequently occurred, have been usually set down, after formal inquiry, to unavoidable accident, when, I have little doubt, they really arose from extreme ignorance or negligence. I never hear of the loss of a transport having either troops or convicts on board, without being very much of that opinion, and for which I will give you some of my reasons. I embarked with General Spencer's force, and sailed from Portsmouth in December, 1808. On Christmas-day it blew a gale from the southward, against which we contended in vain. The men-of-war made a signal, which we afterwards discovered to be that for the first rendezvous, viz., Falmouth; but the instructions of the master of the transport had it not. The flag was there, but the agent had not taken the trouble to have it painted of the regular checquered color, and this was the case with others, so that we did not know it when we saw it. After ten days more contention against the elements, and accompanied only by one other vessel, we bore up of our own accord for Portsmouth; and on the way met a privateer, who ran under our stern, but, finding us full of men, did not like to meddle with us, or we should have found our way to France. This misfortune would have been set down to the elements, or any thing else, rather than to the negligence of the agent of transports, who had not taken the trouble to see that our signals and instructions were correct. He had not, I suppose, any yellow paint, and could not take the trouble to write against the plain flag that its color was checquered yellow and blue. When I came from North America, I kept the ship's reckoning, and took the daily observations with the captain, which indeed I had done before on going out, and we made the Lizard with the loss of only half a day's sail, and afterwards the Start. From this point we took a new departure in the afternoon, and the next morning found ourselves in a fog, by my reckoning, on the back of the Isle of Wight, by the master's, twenty miles from it. As he would not slacken sail, or alter his course, I went forward to look out, and soon saw the breakers. He was frightened half out of his life, but the ship answered her helm, and when we were fairly about, the shore was close under our stern, being St. Catherine's bay, as we learned from a fisherman shortly afterwards, when the fog cleared away, leaving a beautiful Summer's day. When the other officers laughed at him for being beaten by the doctor, he replied, shaking his head, It is true enough, gentlemen, but it all comes of doctoring; which he believed was something like magic, and taught every thing. General Spencer sailed once more from England, and arrived at Gibraltar. Here another accident happened. The St. Domingo, transport, in which I was, anchored on the bank in the bay, and we all went to bed, being in harbour, except the watch and the mate. Being a bad sleeper on board-ship, and knowing the carelessness of the sailors, I went on deck in the middle of the night, and to my astonishment found the ship was drifting. The mate, on being awakened from his nap, under the windward bulwarks of the vessel, would not believe it, until I shewed him the cable nearly right up and down. Well, we turned up all hands, got sail on the ship, and the moment we tacked, the battery at Algesiras opened upon us, (it was before we made friends with the Spaniards), and after some trials, they sent one shot through the accommodation-box on the poop. It made such a noise, and such a hole, that a harlequin from Drury Lane would have been delighted to have had an opportunity of jumping through, if he had been there. We got half-a-dozen more shot from Cabrita point on passing, and then beat our way back, to the astonishment of the fleet and the garrison, who could not conceive what we had been at. If I had not happened to go on deck, we should have awakened in the morning in Algesiras. My last turn in a transport was from Lisbon to Santander, off which port we arrived in the evening, but too late to go in without a pilot; so we stood off and on, and at last lay to, with a good fine breeze on shore; the tiller lashed a lee, and the mate and one sailor to keep watch. At midnight I looked out of my port-hole, and saw the ship was making way fast, and approaching the shore, and there was a very odd noise from time to time on deck, as if the tiller was wagging about at its own pleasure. Thither I repaired forthwith, and found this to be the case. The tiller had got loose, the mate, and sailors were asleep, and the vessel was fast going into Santona, under the guns of which we should have been in another hour. It is therefore with no small satisfaction I have seen that the smaller frigates are to be fitted out as troop-ships. I have heard it said that some of the smaller line-of-battle ships will be equipped in a similar manner; but not, I hope, to carry troops in the beginning of the season, to Canada; for if a 74 should take it into her head to manœuvre against an ice-berg, in April, on the coast of Labrador, she will probably bump; or if she tries a *pas de deux* in the Gut of Canso with another, she will, in all likelihood, make the *faux pas*.

The army landed in Mondego Bay, under the orders of the Duke of Wellington, with the expectation of seeing the enemy forthwith; but no arrangements were made for sick or wounded with the regiments. I went on shore with three days' biscuit in a haversack on my back, which contained besides a pair of shoes, two shirts, two pairs of stockings, washing and shaving apparatus, and a pocket-handkerchief. I was ordered to purchase a mule or an ass for the instruments and medicines, by the commanding officer, who could not give me the money to buy it, nor was any allowance then made for it, as will be seen by the Duke's dispatches. I had two one-handed men attached to me, whose hands I had cut off after maiming themselves in America, and who had hitherto been necessary cleaners,—a class of laborers not required in Portugal. These fellows could saddle a horse or a mule nearly as quickly and as well as if their hands had not been amputated, and having done good service until after Talavera, they retired on a pension for the loss of their hands in action, no one disputing the fact of their having been lost before the enemy. They took care of the jackass that carried the physic, and surgical stores in a biscuit bag, which I begged from the master of the transport, there being nothing else to be had; and thus we set off to fight two or three battles and take Lisbon. By his Grace's dispatches, I see that two bullock-carts were demanded and granted for the medical stores of the army, which, his Grace further specifies, were to carry 25 bearers, one case of utensils, principally, I believe, tin kettles, spitting-pots, and chamber-pots, and one medicine-chest; which specification I know to be critically correct, as the two carts with the above contents, came into my hands at Roliça, to be taken on by me to Vimiera. I have no doubt there were plenty of stores on board-ship, but it is the arrangement, and the utter ignorance of the subject, which is ludicrous. Each surgeon should have had a pair of wicker panniers covered with bull-hide, and duly filled, of a good form and size, slung, and fitted to a proper pack-saddle; and there should have been a horse-transport or two, to carry a horse for the field-surgical stores of each regiment, and from half-a-dozen to a dozen spare ones, similarly accoutred, for extra stores, with a man from each regiment who understood the care of a beast. The extra animals, panniers, stores, &c., might, on landing, have been distributed, one to each brigade in charge of the surgeon best likely to take care of them. It was very unwise to trust to an enemy's country for this supply of animals, and a very paltry economy on so large an expedition. In our own country, such, for instance, as Canada, the horses are the only part of this equipment which ought to be omitted, and they should be furnished by the commissary-general, as one of the first parts of his duty. The allowance made to the surgeon should be sufficient to enable him to keep it up, for it should be an animal more than equal to its work, and should invariably march before the last section of each regiment, and should never be allowed to go to the rear. When we marched to surprise the French, on the advance to Oporto, an order was given for all the baggage-animals to go to the rear. I, however, placed the mule with the physic, and the mule with the entrenching tools, in this situation, and walked and rode by the side of them. In the middle of the night, the general of the division (since dead) passed, and was furiously angry at the order having been disobeyed, and desired to know for what reason I dared to have two mules there. I very submissively replied, that as it was understood we were going to surprise the enemy, it was very probable that they might like to fight rather than be taken, and that we might thereby have some wounded, when the surgical stores would be wanting. He then desired to know what business I had with the entrenching tools. Now I dared not say that such a thing might happen as the pickaxes being wanted to knock down a wall, or for other military purposes, so I very maliciously replied that there might be some killed as well as wounded, and it might be, I thought, as well to bury them. This only made him the more angry, and he ordered them to the rear instanter. In about ten minutes the aide-de-camp came back to say the general would allow the mules to return. As I was quite sure I was in the right, and as events proved it, I told the aide-de-camp they had been gone so long, he had better go and find them, for I could not undertake it; and as the poor fellow saw the folly of the proceeding, he very quietly went to the rear, and brought them back. In this way we began,—how did we finish? When we crossed the Garonne, to fight the battle of Toulouse, the left flank company of the Fusiliers crossed the bridge of boats first, *en tirailleur*, then the band of the Fusiliers, playing the British Grenadiers, then my two mules, as belonging to the chief of the medical staff on that side the water, (and they were the only mules allowed to pass except those of the regimental surgeons,) followed by Sir E. Pakenham and myself. Two French videttes galloped forwards, fired two shots over our heads to announce our crossing, and then retired. Times and men were altered, and it was well known then that a doctor without his apparatus was not much better than a battery of artillery without ammunition. In more peaceable times, the medical staff mules may march with the general's baggage, but when there is something to be done, they should never be out of the doctor's sight; he should be made to keep them equal to any service which infantry can be called upon to do, or mules or horses to perform.

Under these circumstances we fought the battle of Roliça, the surgical part of which, the surgeon of the 9th, Mr. Brown, and I, had nearly to ourselves, for the two bullock-carts and the hospital staff were a long way in the rear. They came up, however, at night, worked with us on the 18th, and relieved me on the morning of the 19th, when I started for the army at Vimiera, with the two carts, and stores in question. I wished the carts at the devil in the first instance, on account of the delay they occasioned; but very soon took a particular fancy to them; for we found the French cavalry patrolling between Lourinha and Vimiera, to the great alarm of both the natives and ourselves. They counted on their fingers two patrols of an officer, and twenty men each, one being before, the other on one side of us, and told us they would cut us to pieces if they caught us. I had some twenty old soldiers with me, all of whom I knew well, and two or three subaltern officers of other corps, who were taking advantage of the convoy, and whose duty it was to fight. I tied the heads of my second bullocks to the tail of the cart which preceded them, and thus continued our march across the country, which was open, although hilly, and soon satisfied my old soldiers that, with their backs against the bullock-carts, they were not to be thrashed by a patrol of cavalry. The pots and pans arrived safe, ready for the battle of Vimiera on the 21st. The 20th was a beautiful day, and I spent it happily with three officers, my messmates, who are all since dead. Captain Gauntlett fell, mortally wounded, on the hill at Talavera, forming the left of our position. He was struck on the side of the head by a ball, which cut his hat, and carried away a portion of his skull and brain. It ought not to have done this, if his skull had been of any thing like an ordinary thickness; it was unfortunately, however, as thin as thick cartridge paper used in packing: it was the thinnest bone I have ever seen, and he lost his life in consequence: he died in my arms in Talavera. Captain Humfrey was struck on the hip by a cannon-shot at Albuhera, which carried away the limbs of two men behind him, and died on the spot, encouraging the advance of the regiment, for the honor of old Ireland, of which he was a native. The third died in bed, Lieutenant-Colonel Stopford, promoted to that rank from home, and unattached; he rode with me the whole fight of Toulouse. The moment the last redoubt on the enemies' right was taken, we rode up to it, and when all was quiet, and I was giving directions about my wounded, the French fired their last shell,—the last, I believe, that was fired in anger against us in France; it burst just over his head and mine. When our first fright was over, he laughingly said, If that shell had killed us both, which of us would have been called the greatest fool? To which I replied, that he would only be called unfortunate, as an amateur soldier, but that I should be called the fool. It is quite impossible for a regimental surgeon to be out of fire, if he does his duty, and a medical staff-officer can scarcely be out of the way of cannon-shot, and the doctors are therefore very unjustly treated in being classed with the clergymen, and the commissaries. They do not actually fight, it is true; but I will venture to say that the surgeons of the Fusilier brigade, in Spain, have been under more fire, take it all in all, than a large proportion of the general officers of the army. I would tell you of the unkindness with which one of these gentlemen is now treated, after thirty years of active service, if I were not afraid my mention of him might do him an injury.

The morning of the 21st dawned upon us in all its brilliancy; distant clouds of dust announced the approaching contest; our breakfast, of biscuit and water, with a bunch of grapes, was soon dispatched; and we at last moved to the high ground on the left of the British position. The fight had begun before we got there, and the French advance was, as usual, valiant. The fire of their guns was heavy while it lasted, and it was at this moment I met with the wounded officer of the 40th regiment, whose case I have alluded to in the 3d edition of my book* whose ball I pulled out of his thigh, for three inches, hanging in his shirt, like a shilling at the bottom of a purse. Whilst I was doing this, he got a crack from a spent ball on his hind quarter, which made him jump, and we thought it advisable to retire into a water-course, which allowed our heads to be under cover. Shortly after this, Sir H. Burrard came on the field, looking remarkably well, as if for a field-day in Hyde-park. I never saw him before nor since, that I know of, nor have I the slightest intention of saying any thing disrespectful of his memory, but that one sight of him quite convinced me he was unfit to command an army in the field. I am satisfied that his head, if examined phrenologically, would have responded to the

* Treatise on Gun-shot Wounds, on Inflammation, Erysipelas, and Mortification, on Injuries of Nerves, and on Wounds of the Extremities requiring the different Operations of Amputation, &c. &c. 3rd Edit. 1827.

best wishes of the Secretary of State who selected him for the service, and that he possessed all the talent a commander-in-chief ought to possess; but he outweighed it below; he was too large in the waist; and although a good soldier may require a large bump or two in the head, he ought not to have them in the belly; and if the Government of this country will allow soldiers to gratify their military propensities, and serve before the enemy, they must take care that they first mortify their gastronomic ones. Lord Byron says, "I hate a dumpy woman;" although the particular dumpy alluded to by him was, I assure you, worth looking at: and I equally hate a dumpy soldier; not that they cannot stand fire, but because they cannot stand fever, at all events I have never been so fortunate as to see one. The Duke of Wellington has several times, in his dispatches, insisted upon it, that general officers going out to Spain should declare upon honor as to their willingness, if not capability, to serve the war. The reverse of this should take place with inferior officers; and if they have any physical disability, rendering them less able and less active than a soldier ought to be, they should not be permitted to go. They should be allowed, if they have deserved it, an honorable retirement; and the 658 gentlemen of the purse-strings should draw them liberally in their favor, if they wish the country to be well served. The country expects every man, before the enemy, to do his duty; but if men are sent, who are physically incapable of doing it as it ought to be done, disgrace and disaster can alone be expected.

General Nightingale's brigade, to which I belonged, formed in line on the brow of the hill, on the right of the position, of the left of the army, with Sir Ronald Fergusson on their left. The French made a show of advancing across the valley, and their officers gallantly set them the example; but it would not do; it was only a straggle, and they soon retired. Our people wese calling for the order to advance; but I told them to keep themselves quiet, for I had over-heard enough of the conversation between Sir H. Burrard and the Duke, a little before, to know there was to be no advance. I had even heard an officer invited to dinner; and I therefore set about my own immediate duty, sure of not being interrupted.

If the affair of Roliça had been highly honorable to the few troops engaged, this had been glorious to a greater number. They were equally important to the advance and improvement of British surgery; which seems to have been utterly neglected in the previous campaigns of Flanders and the Helder. There was a single house in the rear near the front of our position, and I dare say it is there still, which I had selected for such wounded as I could collect of all parties, having few of our own. I had cut off two or three legs with the help of my assistant, when at last in amputating a thigh high up, the tourniquet-buckle broke and it fell to the ground; the bone had just been sawn through, and the limb fell with it; the patient was likely to bleed to death. I seized the bleeding end of the femoral artery with the finger and thumb of one hand, and compressed the artery in the groin against the pubis with the other, whilst my assistant, Mr. Curby, put a ligature on the vessel above my thumb. I found I had perfect command of it with each hand, and when it was secure, and we had time to recover from our alarm, I could not help saying; Why what could Mr. John Bell mean by frightening us all so, by saying it was impossible by ordinary pressure to prevent the passage of blood through the femoral artery? Thinking however I had met with an exception to the general rule, rather than that I had found out the general rule itself, and that Mr. J. Bell might still be right, and I in the wrong, I went into the village of Vimiera early next morning to try the point on another amputation or two. I worked away all day, few people being very gluttonous of work, and established my fact that I was right, and that Mr. J. Bell had given the exception and not the rule. Since that, you know I never apply a tourniquet, for various reasons I have assigned in my work, except when I have bad assistants; and in the latter part of the war, few who knew anything of their profession cared more for any artery in the body, than the troops did for an equal number of the enemy. Both doctors and soldiers thought themselves sure of their opponents. When we came home in 1814, several of us thought we had learned a good deal during our campaign; but as we had not the vanity to suppose that we knew everything, and that our contemporaries in London were standing still; the best of us deemed it right to revisit the various schools at which we had received our early education, and hear what the different teachers had to say. With a great deal of highly useful information, we were surprised also to find that they knew little or nothing of our improvements. In describing the operation of amputation at the shoulder-joint, they could not conceal their alarms; and whilst they were screwing their tourniquets on the shoulders of the young gentlemen they produced as objects for observation, we were internally laughing with all the complacency, and bonhommie their unvaried kindness deserved. I settled this matter by inviting as many gentlemen as pleased to come to the York Hospital at Chelsea, where I had two clinical wards. I took an arm off at the shoulder-joint without any tourniquet or compression at all; and in order that they might be more than convinced, I allowed the great axillary artery to throw its jet of blood over two or three of them, that they might see how easily and completely it was in the power of my finger and thumb.

Sir C. Bell, in his Institutes lately published, has endeavoured to support Mr. J. Bell's opinion, by saying that if he were wrong with regard to the principal artery, he was right in saying that the bleeding could not be stopped because the collateral vessels would bleed, and that a particular compression of the surrounding parts must take place to prevent this. But this is merely begging the question; none of these vessels give any trouble, or are worthy of the slightest consideration. When I come here, gentlemen, and cut off the tip of an old woman's finger, and a thread of an artery throws out its blood, I beg one of you to put your finger on it, that it may not spot my shirt-collar, and frighten the lady I may next visit; and I should do the same with those small arteries coming from the internal iliac in a case of amputation at the hip-joint. They are all in recent cases quite unworthy of your further notice, and in old cases they deserve but little consideration; in fact, as coming from a different source, they have nothing to do with the question. The operation of amputation at the hip-joint was not even shewn, it was declared to be too barbarous to encourage, although we had done it often, and my successful case was actually seen in London, and was afterwards for years the only one that ever had been seen in Paris. An elderly gentlemen sent for me at that time, saying, without further circumlocution, Sir, I have sent for you to know whether you can take my thigh out at the hip-joint. Seeing my patient was an oddity, I replied, I would do it with the greatest possible pleasure, and he desired it might be done at 12 next day. To this I demurred, as it was necessary to know the why and wherefore, and I begged a consultation with the three surgeons whose opinions he had taken. They all declared his case incurable. Mr. Cline pronounced the operation of amputation to be inadmissible, barbarous, and little less than murder. The second surgeon followed his example, and declared he could not countenance it by being even present. The third, Sir Astley Cooper, said, he would assist me if I would do it. It was handsomely and generously said, and as gratefully received, although I did not stand in need of aid, having my choice at that time of my old Peninsular assistants and friends. Under these forbidding circumstances I thought it right to promise the gentleman only one hour to live after his operation was done; that I undertook to guarantee, and on these conditions, we did it. When the operation was done, I said, Sir I am happy to say your leg is off; to which he calmly replied, Sir it was a very unworthy member. I sat by his side for an hour, and when the time was out, I said, Sir, you have outlived the hour, and please God you will recover. Sir, said he, please God or the devil, which you like.

I believe in neither. My trust is alone in you. It was quite clear he was a monomaniac. The next morning he satisfied me however he was mad on two points. The apothecary, Mr. Stewart, came in, and was going to sit down, when he begged him to wipe the chair first, and then spit upon it; and on giving him a pill, he insisted upon the box being shook three times, and spit upon. He was quite sane on all other points, and a remarkably well-informed man on most subjects; but he never could speak of religion without shewing the aberration, nor see the apothecary, to whose profession he had an unsurmountable aversion, without proving it. I foretold, that this operation would nevertheless become too common; that the example we had set would be perhaps unnecessarily followed. I have reason to fear that this has been the case; that I have been prophetic.

After I had done the operation at Vimiera, I have described, and which led to such particular results, I turned my attention to a French officer who was just brought in. He was bleeding much, although not dangerously, from a wound in his face, and his white waistcoat, and trousers were covered with blood. I have alluded to this case in my book as one shewing, that gun-shot wounds will often bleed considerably without any large arteries being injured. I spoke to him in French, he immediately pulled out his little book of accounts, which every French soldier carries, and returned thanks for falling under my care, and gave me his watch and his money as a matter of course, and with the view of begging protection. At the first fight of Roliça, the kit of the dead soldier remained on his back untouched, until he was buried, and it is but just to say, that the British officers taken under the eye of the French General Brennier were not plundered. But our troops were apt scholars, and few dead or wounded escaped plundering after that day, whether friends or enemies; and the progress we made in arts as well as arms, was equally great. The contrast between Roliça and Badajos will be perceived, when I tell you that at day-light after the storming of the town, I saw 13 officers lying dead on the great breach, stripped stark naked in the night by their own friends or their allies. In such a way does war destroy our noblest feelings.

Two days elapsed before I could find time to seek for my wounded French officer, I then found him with several others without a sous amongst them; they had been thoroughly well cleared out, although otherwise well treated, and they admitted they could not have done it better themselves; it was *la fortune de la guerre,* and quite natural. My poor Frenchman came forward to renew his acquaintance, for although I had apparently robbed him, I had still been kind; and kindness begets kindness. You should have seen him open his eyes when I apologized for not having found him before to return his watch and money. They did not believe I was serious, but when they saw me place the watch, and the doubloons in his hand, they could not restrain their feelings. I was an *ange de dieu,* the most beneficent of human beings. I assured them it was only a common act of kindness that every English gentleman would feel himself bound to do. This sentiment seemed however, rather a matter of reproach to men who had avowed that plundering was quite a natural propensity, and they would not admit of it. I was therefore obliged to take my leave, with a full determination to plunder the first Frenchman I came across, in order to satisfy myself, I was but a man.

The convention, so called, of Cintra followed, and gave us all considerable dissatisfaction. A great part of the army never could understand the why, and wherefore, and officers, and men did not fail to express themselves in a very disorderly manner in the hearing of the highest authorities. That they had sold themselves to the French few doubted, although there was not, nor is there the slightest foundation for the suspicion. Years have since passed away, and the propriety, or the reverse of the proceeding has been discussed by various parties, with different political and military views, and feelings. The two principal of them of late date are Colonel Napier, and the author of the critique on him in the Quarterly Review; but neither of them enter into the feelings of the army against their superiors as generally expressed at the time. The discontented officers and soldiers would never have objected to a convention for the evacuation of Portugal, if it had been made and ratified the day after the battle of Vimiera, viz. the 22d, before they knew that Sir John Moore's force could land at Maceira and be in time to assist; but they did strenuously object to its being made on the 28th, when they knew they had that assistance. If the armistice had been broken on the 28th the whole British army of 25,000 men might have advanced on the 30th. Sir Harry Burrard in bringing down Sir John Moore's force to Maceira did better, as it turned out, than if he had landed it in Mondego bay; he gained several marches by it, and instead of that force only reaching Lisbon on the 14th of September, as shewn by the commentators and critics, it might have been easily there on the 3rd. It might have done this even if it had gone by the same road, for Villa Franca on the Tagus is only two short marches from Torres Vedras, and from Villa Franca to Lisbon it is only 21 miles of very good road, with a creek to cross at Sacavem, which would have been crossed as readily in one case as the other. The Duke of Wellington has shewn in his despatches that he would have marched from Sobral to San Antonio de Tojal; Sir J. Moore's force taking the road by the Cabeça de Montechique, on the 31st; and if he had commanded the army and had done so, he would have been in Lisbon on the 2nd of September. We should have had a race for it, but the whole French army left unkilled would have been prisoners, and they would gladly have compounded for the surrender of those at Elvas and Almeida. That Junot might have crossed one-third of his army into the Alentejo is possible, but I doubt it, and he must have sacrificed the remaining two thirds and all his stores and baggage. That the soldiers of the army were right in the opinion they entertained, is quite as clear as that they were wrong in the open expression of their discontent; but they only asked to do what the Duke of Wellington shews he advised, and says he would have done. Although many may doubt his judgment on political matters, few will express it, or even do so on subjects purely military, and no man can be a better judge of what is consistent with the character and honor of a British officer. I shall therefore conclude by supposing that the gentlemen who approve of the convention on the 28th may probably be in the wrong, and that British history instead of defending them, may at least admit, that it is possible they erred in judgment.

Having paid a second visit to Cadiz, which only increased my admiration for the *muchachas muy lindas* of the *Calle Ancha*, the fashionable street of that city, I returned in time to accompany two regiments on a march to Almeida in support of Sir J. Moore. We here performed a piece of animal magnetism, which far exceeds anything the Professors of the present day will I believe ever attempt to do. When we arrived at Castello Branco we halted a day, and therefore had time to prepare our operations. The French had somewhat more than two regiments in Alcantara and its neighbourhood, and this is the essential point for observation, in proof of the superiority of our powers to those enjoyed at present. I believe the principal animal magnetisers do not now claim a power in intensity or force more than equal to twenty or thirty feet, although they can operate on a lady who is duly predisposed at that distance through a door two inches thick, but then only when the magnetiser has his whole mind intently engaged in this one object. It so happened that the minds of the French were as much engaged upon us as we were upon them, and the effect was remarkable, for instead of being bounded by 20 or 30 feet, it extended to as many miles, with a mountain or two between us, and the Tagus to boot, which ought to be as good as a door at any time. Each party turned out as nearly as possible on the second day at the same hour after dinner, drums beating, colors flying, and all prepared for immediate action. No enemy appeared, the uneasiness arising from the extraordinary magnetic influence, which we were so liberally infusing into each other, now took complete effect, and both parties simultaneously turned round and walked off, being quite unable to stand it any longer. The British never stopped until thay arrived at Abrantes. The French took refuge in Truxillo, (pronounce that x as a letter intermediate between it g and h,) and each party only felt themselves free from magnetic influence when they were 150 miles asunder. The day we started, it poured as if the heavens had opened all their watery stores upon us, and no poor devils were ever in a more wretched state than we were on reaching Cernados, one of the most deplorable villages in Portugal. We had intended to cross the Tagus at Villa Velba, but the river had greatly filled in the course of the evening and it was not certain we could cross on the small flying bridge which existed in those days. Under these circumstances we turned short to our left next morning before daylight, and took to the mountains by a bridle road or track, and never stopped until sunset. The bullock cars could make little of this, and half way they were brought to a standstill. What was to be done? After due deliberation it was thought desirable to burn the carts and the baggage. This consisted principally of 20 complete sets of bedding packed in four bales in the hardest manner, and a trunk containing the commanding officer's best suit of clothes, which had no business there. At midnight the conflagration took place. I learned afterwards from a French officer that they were walking off as fast as they could at the same time, under the magnetic influence we were imparting to them, when they saw the light of this fire which they took for a beacon to alarm the country people, in order that they might cut off some of the stragglers. Once he said it seemed to blaze up for a minute or two with more than ordinary brilliancy, which added to their confusion; and he asked what we had done to cause this effect, and did not seem pleased, when I assured him I could only attribute it to the indignant combustion of the colonel's best pair of white leather breeches. Animal magnetism can, you see, do greater things in Portugal than in London.

---

### On Compound and Gun-shot Fractures of the Thigh.

*Delivered Jan.* 6, 1838.

I APPROACH the subject of compound and gun-shot fractures of the thigh with greater diffidence than I do any other in surgery, not from want of experience, or, I trust, of observation on that experience, without which it is of no value, but from the unfortunate nature of the results. Nothing is more easy than to cut this difficulty, by saying, cut off the limb; but amputation is scarcely a less miserable result, and is at all times, even when successful, which in such cases is very doubtful, an opprobrium to surgery.

The best thing I can do, I believe, gentlemen, is to read you what I have written in my work on gun-shot wounds, &c. &c., and then give you the further treatment.

In accidents in civil life, the bone is in general merely broken across, or obliquely, with the point thrust through the soft parts. In gun-shot wounds, it is generally the reverse, being much shattered, and not appearing through the integuments; depending very much on the part of the bone injured, and the manner in which it has been struck by the ball.

If a musket-ball, in passing through the thigh, merely touch the bone, it may fracture it directly across, but it will generally do it obliquely, so as to cause some little shortening of the limb when cured under the most attentive treatment; but when a ball strikes the shaft or body of the femur, it shatters the bone in every direction, although it may not pass through: it does not merely break off four or five small pieces, which may be taken away by cutting down upon the bone, but it breaks it into large pieces, generally oblique and very pointed, that retain their attachment to the muscles inserted into them. The fractures extend far above and below the immediate part struck by the ball; and, as far as depends upon my information from the examination of limbs that were amputated, further downwards than upwards; so that from a fracture in the middle of the thigh, I have often seen fissures extend into the condyles, and cause ulceration of the cartilages of the knee-joint; but they seldom extend upwards as high as the trochanters. Of such cases, there can be no doubt as to the propriety of immediate amputation; but if the fracture did not communicate with the joint, when the middle of the body of the bone is broken into several large pieces, it is better to amputate before the inflammatory symptoms come on, than afterwards; for it must then be done higher up, or probably cannot be done at all.

The danger and difficulty of cure attendant on fractures of the femur from gun-shot wounds, depend much on the part of the bone injured; and, in the consideration of these circumstances, it will be useful to divide it into five parts. Of these, the head and neck included in the capsular ligament may be considered the first, the body of the bone, which may be divided into three parts, and the spongy portion of the lower end of the bone exterior to the capsular ligament, forming the fifth part. Of these, the fractures of the first kind are, I believe, always ultimately fatal, although life may be prolonged for some time. The upper third of the body of the bone, if badly fractured, generally causes death at the end of six or eight weeks of acute suffering. I have seen few escape, and then not with a useful limb, that had been badly fractured in the middle part. Fractures of the lower or fifth division are in the next degree dangerous, as they generally affect the joint: and the least dangerous are fractures of the lower third of the body of the bone. Of these even I do not mean to conceal, that when there is much shattered bone, the danger is great; so that a fractured thigh by gun-shot, even without particular injury of the soft parts, is one of the most dangerous kind of wounds that can occur.

"Upon a review of the many cases I have seen, I do not believe that more than one-sixth recovered so as to have useful limbs; two-thirds of the whole died, either with or without amputation; and the limbs of the remaining sixth were not only nearly useless, but a cause of much uneasiness to them for the remainder of their lives; they were indeed much in the same state as Bilguer's invalids, who were incapable of any employment, civil or military.

"It would be an interesting, and I am sure a useful inquiry, to examine the lists, or cause lists to be made, of British soldiers who receive pensions on account of incapability for service, from wounds with fracture of the thigh-bone; and I am satisfied the number would be small, although the accident is not infrequent; and of the number thus receiving pensions, I will venture to predict, it will be found that in seven-eighths the bone was broken below the middle of the thigh.

"After the battle of Toulouse, forty-three of the best of the fractures of the thigh were attempted to be saved; having been carried from the field of battle but a very short distance, well accommodated in hospital, and attended for the most part with great care, and surgical attention: of this number, thirteen died; twelve were amputated secondarily, of whom seven died; and eighteen retained their limbs. Of these eighteen cases, the state, three months after the battle, was as follows: 'Five only can be considered well, or as using their limbs. Two more think their limbs more valuable (although not very serviceable) than a wooden leg: and the remaining eleven wish they had suffered amputation at first, as they are not likely to do well; and if they eventually recover, which in many is doubtful, the limb will be distorted and unserviceable.' Of two officers with fracture of the femur, one died in the hands of the French surgeons, in whose charge he fell during the action, and by whom he was skilfully treated; the other, with the greatest possible attention and care, has preserved a limb, which I think he now wishes exchanged for a wooden leg.

"In the five successful cases, the injury was, in all, at or below the middle of the thigh. In the thirteen others, who retained their limbs, the injury was not above the middle third; and of those who died unamputated, several were near, or in the upper third, and either died before the proper period for amputation, or were not ultimately in a state to undergo the operation. Of the seven amputations that died, two were at the little trochanter by the flap operation, and the others, for the most part, unfavourable cases. In one case only was the head or neck of the bone fractured by a musket-ball, which had entered on the outer and back part, and went through in front. This man was not pointed out to me for some days, and was not at that time, or ever afterwards, in a state to render amputation likely to be successful. He lived however for two months; and, from the dreadful sufferings he endured, I always regretted amputation at the hip-joint had not been performed at first.

"After other battles, in which I have had the care of fractures of the femur, the success has not been so great, but they were generally under less advantageous circumstances; and from the sum of knowledge thus acquired on many occasions, I am induced to believe, that in this injury, amputation ought to be a more frequent operation than it is at present; and I think I am borne out in this supposition by the above statements, and by the general opinion of my brethren formed during the Peninsular war.

"I think it will also be conceded by those who are disposed to allow the advantage and safety of primary operations, that if the thirty-six of the forty-three who died, and have only partially recovered, had been amputated on the first day, the country would have had at least twenty-five stout men, able, for the most part, to support themselves by their labour, instead of five, or, at most, ten, who will not be entirely dependent upon their pensions and parishes for their subsistence.

"As secondary amputation is totally inadequate to produce this effect, the patient should be carefully examined, and amputation performed, when necessary, on the field of battle. If the heat of the weather be great, as in the Summer of the Peninsula, Asia, or America, the hospital to which the patient must be removed at some distance, the means of conveyance bad, or the wounded very numerous, it is better to amputate, even in a doubtful case; and if the surgeon, by following this rule, should even cut off a limb that might have been saved, he will be amply compensated by the preservation of a number of lives, that would be lost by delay under precisely similar circumstances.

"In regard to officers, some little more latitude is to be granted than the above suggestions allow; for, as they can often procure cool apartments in Summer, good conveyance, plentiful attendance, and the best professional advice, all of which are occasionally wanting to soldiers, cases of disease and injury will always succeed in a greater proportion with them than with private soldiers in hospital; but not in so great a degree as to counteract my opinions in cases that are really serious.

"It is a difficult thing to persuade a surgeon unaccustomed to the treatment of gun-shot wounds, or the patient himself, when he sees but a small wound, that amputation is necessary; and as cases of success have been heard of by all, whilst the fatal ones are buried in oblivion, many officers will not choose to submit to it; they will rather hazard their future health and happiness, and undergo the most dreadful sufferings, for months, to save a limb, which, when cured, and their wishes are obtained as far as circumstances will permit, they find a useless burthen, and a source of inconvenience for the rest of their lives."

Wounds from musket-balls, injuring the lower part of the bone, without communicating with the joint, do not require primary amputation; they are proper cases for delay, unless there be great destruction of parts.

In order to attempt, with a reasonable hope of success, the management of a compound or gun-shot fracture of the thigh, it is desirable the patient should be placed on a proper bedstead, of sufficient height from the ground to render him easy of access, and capable of affording him every necessary comfort and accommodation without moving. This will be best accomplished by a bedstead and mattrass, invented by the late Mr. Gardiner and myself in 1815. The one he died upon in 1817 is now at Mr. Knox's, 107, Jermyn-street. Mr. Gardiner, if alive, would have been now Lord Mountjoy, and I am disposed to call the bedstead by that name, to distinguish it from all others. It is so absolutely necessary in cases of diseased hip and spine, of loss of the use of the lower limbs, of injury of the pelvis, as well as in fractures, that one or more ought, in my opinion, to be placed in every permanent hospital in the British dominions. There is one now in use in the Westminster Hospital, and it has been the comfort and happiness, as well as the saviour, of more than one person who has lain upon it. I am aware of the difficulty of carrying these bedsteads to the scene of action in a distant country; I am equally aware of the expense: but Great Britain must give up all her pretensions to humanity, if she allows either of these trifling obstacles to prevent her brave defenders from having an absolutely necessary assistance for their recovery from their injuries, or for the safety of their lives. A bedstead, mattrass, &c., complete, may be easily bought, packed, and conveyed to the most distant of our possessions, for ten pounds; and six of them may be carried upon any common cart to wherever they are wanted. If a second inclined plane and another set of bedding are added to the box, it will cost thirteen pounds, and will answer for two fractures. The battle of Toulouse gave 43 fractures of the thigh, out of 1242 wounded, which were attempted to be saved, and this may be considered as a fair average; and I should say that a corps of 10,000 men ought to be supplied with a reserve store of at least 20 of these double-bedsteads, at an expense, perhaps, of £260. In Spain and France we had nothing of the kind; the consequence was, that many suffered intolerable torments, that might have been greatly alleviated, and many lost their lives. Not only that, many could not be attended to, from the delay and the difficulty of assisting them on the ground; and it is only necessary to have seen the horrible sufferings they endured, and which might have been relieved, to speak strongly, as I do to you. I wish it to be received as a voice from the dead, calling upon the gentlemen of England, upon this, and upon all other points, to do one of two things—to send their sons forth to fight, with every hope, and every surety that if they fall wounded in the service of their country, they will have every attention and comfort afforded them that the talent, the capability, and the money of the country can bestow; or not to send them to fight at all. The Duke of Wellington, in his despatches (10th vol.), has expressed his opinion of the late Medical Board in no measured terms; but the present one, with all the knowledge and all the persevering labour of its able Chief, can do very little better, as far as regards the exigencies of the sick and wounded soldier, unless assisted by the Treasury of the country in a very different manner to what has hitherto been done. I beg I may not be misunderstood. I do not want more pay for the medical officers of the army, the Government has acted liberally towards them in that respect; but I want almost every thing else, both for them and for the wounded. The misfortune is, that things in this country do not go through one channel, and unless one or two powerful men, or several smaller ones, will take up a question of this kind, it never can meet with the consideration it requires and deserves, in all the various quarters with which it is connected.

The position of a fractured femur must be essentially of one kind, viz., straight; for it is impossible to keep a man's thigh in the bent position on the side, and himself in the same situation. No power that is likely to be employed can prevent his turning on his back, and the union, if it takes place at all, must then be at an angle. The bent position forwards, or on an inclined plane, is defective, inasmuch as the matter, which must necessarily be secreted in abundance, will gravitate backwards, in spite of every care to prevent it; and in many instances will form abscesses towards the pelvis, instead of always running directly outwards by a fair, and unobstructed passage. When a proper bedstead is used, a slightly inclined plane may be tried at a certain later period of time, and in some few cases the body of the patient may be raised even to the erect position. This must be done however with care; the object is to take off the action of the two muscles inserted into the little trochanter, which raise the upper end of the fractured bone upwards and outwards; which you will invariably see take place after every ampuation as high as the middle of the thigh. In simple fractures position will do this, and the inclined plane, whether by splints or other machinery, will effect it very well; but as an inclined plane can be rarely used with advantage in gun-shot fractures, the rising end of bone must be kept in its place principally by raising the body or by proper and well-directed padding; and its inclination outwards must be met by a similar direction of the lower part of the limb. The advantages I have alluded to, the army never hitherto had, nor any proper splints, nor any thing which could be called proper for the treatment of such fractures; but they will all, I feel assured, be in future supplied, and you will have the opportunity of ascertaining facts upon this point which will be of the greatest advantage to mankind. I point out how far we have gone before you; it is for you to shew by how much you can exceed us.

Aware, from what I have said, of the nature of a gun-shot fracture in the arm, you know what you have to expect in the thigh, and must endeavour to meet it. First, by the removal of splinters, and extraneous bodies. The bone being larger than that of the arm, the splinters are often more numerous and larger, upon which depends the question of amputation; but that having been decided in the negative, they are to be treated in a similar manner, and the splinters, and extraneous bodies must be as far as is possible removed. It must be borne in mind that they can never (I shall give the general rule) be all removed at once, or at the first, or even succeeding examinations; and it follows, that as they cannot come away of themselves, except they are small, incisions must be made for their removal, and before any quantity of new bone can be formed around them. This is a difficult and very interesting point of practice, which observation will render more clear to you. It is sometimes neglected, from the great thickness of the muscles of the thigh, and from the wound having been on the inside, near the great vessels, so as to render an incision of sufficient size in some degree dangerous. The thickness of the muscles does not offer a sufficient reason for avoiding an incision; and if the situation of the bone on the outside of the thigh be clearly known, a little reflection will shew that it can be got at easily in that part, if it cannot in another. The bone I now shew you is the crooked one I have alluded to from Albuhera. It has united at an angle, with a deplorable twist, on account of the wrong position in which the patient lay, and from the want of every proper means. That is, however, the least part of the evil; for a man can live with a crooked thigh; but the new formed bone is also obvious, and in various parts it hems in or surrounds several splinters of old bone, which are thin and small, although long, and could not get out. They therefore kept up an irritation which at last killed the patient at Elvas. The best cured fractured thigh I know of from Albuhera is Lord Ventry's. It was broken rather above the middle, but he is lame, and it even now gives him some trouble. The thigh-bone I now shew you is from Waterloo; it is a much better one, nearly straight, and the splinters were nearly all removed; but it killed the patient nevertheless. If he had had all the comfort and acommodations he would I hope have had by modern appliances, he might have recovered. The third thigh-bone I shew you has evidently been amputated high up. It is one that was cut off after the battle of Vittoria. The bones have united by a mass of new formed ossific matter, which is hollowed like a scooped-out orange rind. There is a long splinter of old dead bone locked in by it, and lying across, which kept up irritation, and ultimately led to amputation. It should have been removed at an early period. Even amputation, under these circumstances, does not always give relief, or save life; for, independently of the hazard of this operation high in the thigh, to which I have sufficiently alluded, the femur above the injury is not always sound. It is suffering from some irritation of its internal membrane, and the saw only adds an injury, which causes an increase of mischief. The internal membrane dies, and a necrosis takes place for several inches upwards, even as high as the small trochanter. Here are three specimens of this disease after amputation. You see the sawn part quite sharp at its edge, shewing that it lost its life forthwith, that the absorbents did not act at all in rounding it off, as they do in successful cases. The five or six inches above are in the rough worm-eaten state of a sequestrum. It is the old bone, which has come out of a new case, formed by the periosteum around it. Although this state of evil occurs after gun-shot fractures, I am aware that it can also take place after amputation from more common causes; and I have therefore warned you against the old practice ot scraping the periosteum from off the bone at the part where you are going to saw it, or of making two or more jags with the saw in consequence of its slipping. I have told you that 70 to 80 seconds is enough of time for cutting off a thigh; but then you must know how to do it. Sixteen seconds gain of time to you in sawing a bone, may be sixteen months of misery to your patient; let time therefore be a secondary consideration. If you think, gentlemen, you can saw bones by intuition, you will err; it requires practice, like every thing else. If you wish to learn how to saw the bones of living men in an artistlike manner, buy a couple of broomsticks, and saw them off by inches, under the directions I have given for the management of the saw; you will then be qualified to try your hands on dead bones, and after that on living ones.

It is possible to succeed in saving thighs fractured any where except at their extremities, by attention to all these things, if you have at hand all the necessary appliances of surgery; but if you have not, and the sufferer must be kept in a constant state of motion and of irritation, you had better cut off his thigh; or, as a brave, but unfortunate, French officer, said to me at Salamanca—Pray Sir, kill me in any way you please, but do not, in mercy, let me die by inches.

Splints for fractured thighs must be of three kinds, with proper pads for all of them, and the material to make the pads should accompany them in considerable quantity.—1. Long Dessault splints improved. 2. Amesbury's leg and thigh splints, to be used straight for the thigh. 3. Common solid wood and tin splints. 4. Ordinary one yard roll-up measures to take the length of the thigh and leg from time to time; although, in compound fractures of the thigh, extension is of little or no use at any time, and of none after the first fortnight or three weeks; on account of the consolidation, and thickening of the soft parts, which after that time do not admit of any yielding, although the bones may not be united by ossific matter.

### On the Excision of the Head of the Thigh-bone.

*Delivered January 13th, 1838.*

When the head or neck of the femur have been injured by musket-balls, the patient has never escaped with life to my knowledge, unless after amputation. He does not die however immediately, but lingers for two, three, or even more months, and at last sinks worn out by suffering. The bone I now shew you is that I removed in my successful amputation at the hip-joint after Waterloo at Brussels. The ball struck the neck immediately below the head, and went through and out behind; the bone is split nearly perpendicularly, and is separated from the shaft by a triangular point or apex exactly two inches below the small trochanter. The great trochanter and the rest of the shaft were not broken. I am not sure that if the head and broken piece had been removed and the shaft sawn straight, he might not have recovered; as he can however walk very well, I suppose he is as well satisfied that he did not take the chance. There was one other case at Brussels, in which I wished to do this operation. I urged it on the man, a French soldier, for several days, but he refused until finding himself sinking, when he consented. It was too late; when I gently stated this to him, he thanked me, a tear for the moment glistened in his eye, he waved his hand once more over his head, and cried out, *Vive l'Empereur*. He died a few hours afterwards.

The bone I now shew you is one to which I wish to draw your particular attention. The ball entered high up on the thigh near the groin, and lodged in the neck of the thigh-bone; it gave rise to ulceration of that part, of the head, and of the shaft near it, but it did not break or splinter the bone itself; the man however died. The removal of the ball alone would not have saved him, but I am satisfied the removal of the head and neck of the femur might have given him a better chance than he had by doing nothing.

The third and fourth preparation I now lay before you, shew a greater degree of mischief, and a larger quantity of new-formed bone, indicating that the fracture had extended into the trochanters. In the fifth preparation, the fracture is too far below the head and neck of the bone to admit of any operation but that of amputation.

I have been long acquainted with a nobleman, who was sent to Lisbon by Mr. Hunter near 50 years ago for ulceration of the cartilage and head of the thigh-bone, which eventually was drawn out of the socket as the capsular ligament gave way, and has formed an attachment with the ilium. The joint is stiff, the leg is some three inches shorter, but he walks about on the toes of that side with no further inconvenience than the limp occasions.

Mr. White, my colleague at this hospital, removed the head and neck of the thigh-bone in a young person, by a very simple operation, after it had been dislocated by the same disease, because it was evidently causing great irritation, instead of forming a false joint as in the former case, and would eventually have found its way through the soft parts. The lad recovered.

A due consideration of these cases led me to think that if Nature could, by certain slow processes, arrive at such a happy result, it might be possible, in gun-shot fractures of the head and neck of the bone, to give her that assistance by art in effecting a cure which she has hitherto seemed unable to obtain without it. This assistance is the early removal of the head and neck of the bone by sawing it across immediately above the small trochanter in all cases in which the nature and extent of the injury will admit of such an operation. In the bone I now again shew you, and to which I have already drawn your particular attention, this might have been done with great ease by the surgeon, and I believe with little comparative suffering to the patient.

In order to do this operation with precision, the surgeon must make himself well acquainted with the anatomy of the parts by dissecting them himself. I have requested the demonstrators of anatomy in the school of the hospital, Messrs. Hancock and Hird, to prepare two limbs duly injected, to shew it to you. One is dissected so as to allow every part to be demonstrated and divided in the clearest manner. Mr. Hird will have the goodness to divide as I name them. Observe, in the first place, two things, the great trochanter, and the round head of the bone in its socket, which is directly below, and a little internal to the anterior superior spinous process of the ilium. When the thigh is bent in the dissected state you see it rolling very distinctly; and in order to lay it bare for removal, the muscles, &c. around it must be divided. The first on the anterior and outer part is the tensor vaginæ femoris; you see Mr. Hird now dividing it; outside this the glutæus medius must be cut, going to be inserted into the upper and outer part of the top of the great trochanter; deeper and between these two last, you see the glutæus minimus winding forwards to be inserted into the anterior portion of the same part. Cut the great glutæus through backwards in a curve, and you see the insertions of four muscles at one part, viz. the pit or fossa immediately behind the great trochanter, (I now shew it to you in the dry bone); these are the pyriformis, the gemelli reckoned as one muscle, the obturator externus and internus; they should all be cut through within half an inch to an inch from their insertion. The square muscle you see below them, running from the ischium to the inter-trochanteric line I now shew you is the quadratus femoris. It cannot be spared. The thigh-bone now rolls in Mr. Hird's hand easily, and you see the head moving in the socket on the back part where the small muscles were divided. He now opens into the joint with the greatest ease at this part, and by a little rotation of the knee inwards, the head of the thigh-bone is readily dislocated downwards and outwards. The round ligament, and the remainder of the capsular ligament, must now be divided, keeping the knife close to the bone. You have now every thing exposed that I should have wished to remove, and ready for the saw, in the particular case I have shewn you, and to which I have considered this operation so applicable. Let us pause a moment before we apply the saw. Two strong muscles are inserted into the small trochanter, the iliacus internus and psoas magnus, and I am desirous that this insertion should remain unhurt if the fracture should not extend below the little trochanter. It is not necessary to injure them, and they may be of great use afterwards if the operation should prove successful. If the neck of the bone is broken through, rotating the thigh as I have directed may not assist much in dislocating its head; but then, if the separation is complete, the separated piece can readily be detached, when the object will be easily attained. The sawing is as you see accomplished with the greatest ease. The arteries divided are all of small size; they are filled with red injection, yet you can scarcely see them, and they could not give any trouble, for the wound is so large as to give easy access to every part, and to admit of any bleeding vessel being tied without any difficulty. The round ligament should be cut off close to the socket, and any portion of the capsular ligament which can be quickly removed with it, but no time should be lost in trying to remove the cartilaginous cavity itself, which will be gradually absorbed. The sawn end of the femur should now be brought up into the cavity, and kept there if possible with the hope that it may become rounded, and adhere by a new-formed ligamentous structure in the same manner as the end of the humerus does to the glenoid cavity of the scapula, when similarly treated. The edges of the wound are then to be brought in apposition, and retained so by two or three sutures. The glutæus magnus slides over the trochanter major, having a bursa between them, and this part will not readily throw out healthy granulations; I am therefore less solicitous about the accuracy of the apposition of the edges at the under part through which the discharge will the more easily pass; the outside must however be supported by sticking-plaster and a compress to prevent any bagging, and to keep all parts in contact.

Let us now do the operation on the undissected opposite limb. The first cut through the skin or integuments and fascia lata should be a curved one, beginning just over the inner edge of the tensor vaginæ femoris muscle as you have seen on the other leg, curving downwards and outwards, so as to pass across the bone an inch at least below the trochanter major; when it should curve upwards to the extent of three inches or more, as the size of the limb may require. This first incision should, when complete, divide in addition to the integuments, the fascia lata, the tensor vaginæ femoris, the glutæus medius and minimus, and the great glutæus muscle. The flap thus formed must be raised or turned up by an assistant to enable you to get at and divide the parts below in the order I have named them before. You are not to stop to tie any bleeding vessel until the operation is finished, and little or no blood will be lost.

Let us pause again. You have just done one half the operation as to cutting, for removing the whole limb at the joint: and if you should now find that the bone is so much shattered in the shaft, that you cannot hope to save the limb, there is no difficulty in removing it. Place your long knife inside the bone, with the middle of its edge resting against the outer edge of the iliacus and psoas muscles, and at one firm cut of a strong hand let it cut its way inwards, forming an inner flap; your assistant steadily compressing the femoral artery against the bone above. The femoral artery and its great profunda will both be divided: you seize them with the finger and thumb of your left hand, and place a ligature, or assist in placing one on each branch with your right; or, if the trunk of the profunda should have been cut very short, you may tie the main trunk of the femoral. Let your ligature be a single thread of strong dentist's silk, with which I have successfully tied the common iliac, and you need not be afraid of its not holding fast, if you tie it reasonably tight. The idea usually entertained, that a great artery cannot be closed by the ordinary process of nature under a ligature, if a branch is given off near it, is, I believe, erroneous. I never placed reliance on this opinion, and I now shew you the common iliac artery of one of the two cases in which I tied it successfully, the patient dying a year afterwards, and you may see it is tied about an inch from the aorta, and was pervious on each side of the ligature, which has closed the vessel to no greater extent than its own width, proving all the facts I have mentioned to you so frequently on this subject. As to the smaller vessels, they will give you no trouble, being easily commanded, each by the point of a finger.

I have not done this operation on a living man, but *you must do it*, and I am sure you will in the end succeed. You ought not to be allowed to take out a limb at the hip-joint, unless the head and neck of the thigh-bone are injured; and you ought not to take it out if they are, unless the shaft of the thigh-bone is irreparably implicated also. In all such cases the inspector-general should see the bones, as well as have the other particulars of the case. I give, gentlemen, the experience of former times, matured by the practice of twenty years in this hospital, and by a due consideration of all that has been done by my contemporaries. It is for you to surpass us, to shew that surgery is never stationary, and to prove that it is as much a science as an art.

The fifth portion of the femur, or that which forms its lower end and condyles, entering into the composition of the knee-joint, is the least amenable to surgical treatment. In the present state of our knowledge, and after the trials which have been already made in vain, I do not recommend the excision of the knee-joint, and amputation is then the sole resource, in cases which are incurable by ordinary means. I have endeavoured to bring under your observation those cases which generally require amputation when the knee-joint is injured, and those in which an attempt ought to be made to save the limb. See page 379, *et seq.* of my work on gun-shot wounds, to which I must refer. The lower part of the femur, just above the condyles, admits of a particular injury, from its softness, which does not occur at any other part. A ball may pass directly through, without causing any other injury, or scarcely any longitudinal fracture; so that the sufferer walks after it as if the bone had not been broken. Captain St. Pol, of the Fusiliers, was wounded in this manner by a musket-ball at the foot of the great breach, on the night of the assault of Badajos. You will find his case, terminating in death, page 237 of my work on wounded arteries, to which I refer you. The ball struck him in the ham, passed through the thigh-bone in the middle, just above and between the condyles, and fell into the cavity of the joint, from whence it was removed. The hole in the bone was only large enough to allow the ball to pass through, and a small fissure extended upwards from it for above three inches. He died from mortification, the popliteal artery having been also divided. I fear he lost his life from excess of kindness; for the surgeons of his regiment were much attached to him, and I knew him very well, and yielded to their solicitations to try and save his limb, or at least give him a chance for a secondary amputation. They did this against their better judgment, and so did I. It was the last case in the Peninsula in which I did not insist on immediate amputation, on the occurrence of gangrene after a wounded artery, although I admit it was not the last in the army, and that one even of a similar nature occurred at Toulouse, but against my express orders. His Majesty Louis Philipe, King of the French, when he was in England, during the French Revolution, studied mathematics it is said, with great success, and taught them in a very satisfactory manner. Continual occupation in squaring a circle, however, could not but be onerous; it was "toujours perdrix;" and His Majesty relaxed a little occasionally in other less severe studies of nature, his capabilities in which, and in calculating nativities, were soon exemplified, it is said, by the appearance of poor St. Pol. I do not know whether he was like him, but he was a very handsome, agreeable young man, who conducted himself always like a gentleman and a soldier, and died much lamented.

On the subject of fractures of the leg I must refer you once more to my work on wounds, begging you to recollect that all I have said in these lectures with reference to the arm applies as much in all practical points of surgery to the leg. The bones are equally exposed, the vessels are equally at command and under control; and a man should hazard a great deal before he should lose his leg, if he has a good constitution. I do not say to you that you should never amputate a man's leg on account of a musket-shot, which fractures the bones, injures the arteries, and does other mischief; but I do say that it is a very great extent of mischief alone that authorizes such a proceeding. You must read the passages I have written on this subject, and from page 257 to 261 in my work on wounded arteries, and operate as directed from page 384 to 388. I am aware that these things are but little taught in London, or, if they are taught, they are little attended to; but they are essential, and every man ought to know them, and he who does not is not qualified to hold Her Majesty's commission in the public service of the country. When I say these things are little attended to, I do so from finding it to be the fact in the examinations at the College of Surgeons. One of the last men I examined on these points was himself what is called a grinder, viz., a gentleman who crams others for the examiners, as old wives in the country do turkeys before Christmas for the Cocknies. He knew little about the matter; but as I am always particularly lenient upon any point which I consider peculiarly my own, I let him examine me, instead of my examining him. It did just as well, and I dare say he will remember it longer.

When balls impinge with sufficient force to stick in a bone without splitting it, or go directly through, a great deal more attention should be paid to them than was generally given during the Peninsular war. This is one of the weak points on which we wanted another campaign in the South of France to perfect us; and it is therefore one to which you, gentlemen, if there should be, and when there shall be, another war, must attend to particularly. They should always be removed if possible; certainly so if the approach to them does not cause more danger than leaving the ball to the operations of nature. I have known balls stick in bones, and become covered up, and give no trouble; but I have very, very much more frequently seen the reverse. When a surgeon is acquainted with the anatomy of the parts, he is not afraid of making an incision of a sufficient size to enable him to feel, and perhaps to see the ball. If it is deeply imbedded, a trephine must be applied over it of such size as will include a common ball a little flattened. Where it sticks without being imbedded, a small chissel, straight or curved at the end, or a hook used as a lever, may often be got under it; but the judgment and ingenuity of the surgeon must be exercised on such occasions. Suppose a man to have received a ball, which sticks in the outside of the lower end of the femur above the joint: is it better to leave this to the efforts of nature, or to take it out? If left alone, the man will, in all probability, have an open wound for years, suffer much occasional pain, have an abscess form now and then, and always have a reminding monitor of his wound along with him. If a sufficient incision, on the contrary, be made at first, on the day he receives the shot, through the vastus externus, down to the bone, and extending upwards far enough so as to allow it to be seen, whilst the parts are not adhering, and will separate easily, the trephine may be applied without difficulty, or the ball may be elevated and removed. The bone will not be diseased, and will in general recover from the injury without difficulty, and any apparently splintered or injured parts may be at once removed. The man will soon get well, and be less lame than if no operation had been done.

Suppose the ball has struck the pelvis, or hip, just above the acetabulum, and sticks—what ought to be done? Make an incision upwards and outwards, not inwards, and of sufficient length to enable you to feel it easily, if not to see it. You must then remove it by similar processes, recollecting the thickness of the bones of the pelvis, and where they are too thin for much use of the trephine. Of balls sticking in the bones of the cranium we shall speak in our next lecture.

I cannot conclude this subject of injured bone without drawing your attention to three cases now in the hospital, two under Mr. White's care, one under mine. The first was a bad case of ulceration of the tibia, which had gone on for three years, and seemed likely to go on for ever, unless some more vigorous mode of treatment was had recourse to than had hitherto been adopted. Mr. White applied one part of the chloride of zinc, mixed with two parts of the sulphate of lime, made into a paste with water. The diseased bone was in part destroyed by this caustic, and a thick scale, the size of the paste, was separated, and came off. The first application was made on the 14th of October, and a second was had recourse to on the 7th of November, with the same effect, the scale of bone separating in fifteen days. The ulcer is nearly healed, and the man is almost well. In the second case, the tibia had been diseased for 31 years; the mischief was so great that the man begged to have his leg cut off. The repeated application of this remedy has been so effectual as to leave no doubt in my mind of its great utility in similar but less extensive injuries, for although it has rendered him a very essential service, I am not quite certain it will be able to effect cure, nevertheless, it will prevent I have no doubt, the necessity for amputation. In the third case, which is that of a woman, the injury of recent date, and I have applied it solely for the purpose of causing the separation of a thicker scale of bone than is exfoliated by the unassisted operation of nature, and I recommend it to your attention in cases of ulceration of bone, commonly called caries, as one from which you will find the greatest advantage.

### On Excision of the Elbow-joint.

*Delivered February* 10, 1838.

I have pointed out to you so distinctly the manner of excising the head of the humerus, that I do not think it necessary to notice it here. The excision of the elbow-joint I think it right to demonstrate, and to describe to you more clearly, so that you may find no difficulty in doing it.

The cases which require this operation are those in which the articulating ends of the humerus, radius and ulna, are wholly or in part so much injured, that little or no hope remains of a successful result. These cases have been usually submitted to amputation, on account of serious injuries of joints being rarely cured. The object of excision is to save the fore-arm, or at least to give the patient a chance of doing so; and the situation of the shot-hole or holes does not signify much as to the manner of operating. The principal point regulating the proceeding is to preserve the nerves entire; and the most important one likely to be injured is the ulnar. To avoid this, which lies between the olecranon and the internal condyle, but nearer to the inner condyle to which it may be considered to be affixed in passing by; a common straight, but strong-pointed knife is to be pushed into the joint, immediately above and close to the olecranon process, exactly at its inner edge, near to, but at a safe distance from the ulnar nerve. The incision, thus begun, is to be carried outwardly to the external part of the humerus, dividing thereby the tendinous insertion of the triceps. From the end of this transverse incision a cut is to be made perpendicularly upwards and downwards, about an inch and a half each way, and the same is to be done at the other end of the transverse cut. The whole will now resemble the letter H, and the two flaps thus formed must be turned up and down. The olecranon may now be readily sawn off, with the great sigmoid cavity of the ulna, and the coronoid process, which must be separated from its connexion with the brachialis internus muscle which is inserted into it. Before this is done the ulnar nerve must be carefully separated from its attachments and turned aside that it may not be injured. This exposes the joint effectually; and the head of the radius may now be cut through by the saw, or large scissors in cases of old standing disease, and separated from the lateral ligament and the humerus, care being taken not to cut, if possible, the insertion of the biceps into its tubercle. The articulating extremity of the humerus ought now to be pushed through the wound, and the broken end sawn off or removed, so as to leave it quite smooth. The brachial artery and median nerve are out of all reasonable distance for injury, and to prevent the possibility of its occurrence, a knife or a spatula may be placed underneath, close to the bone and quite across, before the saw is applied; and any vessel which bleeds freely may be tied, although it is probable the hæmorrhage will cease on the application of cold water. The articulating cartilaginous broken ends of the bones having been thus removed, and the hæmorrhage having ceased, the fore-arm is to be bent, and the ends of the radius and ulna are to be brought in apposition with the extremity of the humerus. The incisions are to be brought together by stitches and sticking-plaster, duly supported by compress and bandange. Mr. Syme, who has paid great attention to, and has earnestly recommended this operation, strongly advises that strict attention should be paid to procure union, if possible, of the transverse incision, just above the olecranon; for a broad cicatrix interferes with the motion of the joint, a reasonable degree of which is always to be expected in a successful case. The arm should be duly supported by a proper sling. As the shot-holes must remain open, any discharge of blood or serum which takes place will readily pass through them. The first dressing should be early changed, and the incisions kept in due apposition, so as to offer every chance of union; but I do not think this of so much importance as has been attached to it. Passive motion should be early given to the parts, so as to favor the formation of a false joint; but this should be carefully and moderately done, lest too much excitement should take place, and a new inflammation be excited. A fixed or stiff joint is not to be desired, but if it cannot be avoided, it must be procured with the fore-arm at a right angle with the arm, when it will be most serviceable. The patient is best placed on his face on a bed or a table, which renders the steps of the operation more convenient to the operator; you must also recollect that when the bones are diseased from inflammation of any kind, they become soft and are readily cut with the strong scissors, but when they are sound, as in cases of gun-shot wounds requiring this operation, they retain their natural hardness.

---

At San Antonio de Tojal, a circumstance occurred which it will be useful for those to know who may serve in hot countries. A soldier had suffered from bleeding for several days, in considerable quantity, from the mouth, which was perpetual; some was spit out with a little cough, some was swallowed, and neither medicine nor treatment seemed to have any effect upon him. He became greatly emaciated, and his death appeared to be inevitably at hand, although I could not discover any particular disease about him. On visiting the hospital early in the morning, I inquired if he was dead, and was astonished at being told he had been quite well for two hours, and intended to live, for that he had coughed up a leech, which there could be no doubt was the cause of the bleeding, inasmuch as it had ceased from that moment. The man rapidly recovered. I was quite aware that in warm countries, in which leeches prevail, they are readily taken up by men and horses in drinking out of puddles, as thirsty animals, whether bipeds or quadrupeds, will constantly do. They are usually what are called horse-leeches, or of that kind which hold on and suck at one end, and discharge the blood at the other; but they commonly stick about the lips, mouth, and throat, both in men and horses, from whence they are readily removed by the fingers or forceps. When they get above or behind the palate, they are still usually discovered with a little trouble; and when they could not, I never before or afterwards found much difficulty in dislodging them with strong salt and water injected through the nose, which, by its own virtue, and that of vomiting, had the desired effect. Whether this leech was in that place or not I do not know, but it certainly did all but kill the man.

Dr. Robb gave me the particulars of a case, which was afterwards confirmed by Mr. Maling, who said he saw it, which occurred in the light division, and nearly killed a man, who had been drinking out of a puddle, or out of a canteen filled from one, in a more extraordinary manner. The man declared he felt something move in his stomach the instant he had drunk, and from that moment his torments were unceasing, both from pain and from the alarm he felt at distinctly perceiving something trotting up and down his stomach. The man became pale, wan, and miserable, and would have died, in spite of all the means employed for his relief, if he had not one day, at the end of about three weeks, vomited up a living animal, the cause of all his misery. The case is so well attested by the medical officers, who saw the animal before it died, that it cannot be disputed. They say it had four feet and a long tail, and called it a salamander, I presume from no medicine having had any effect upon it, rather than from its really deserving that name. It was so large that it nearly choked the man in coming up, so that he was quite satisfied it had grown considerably after he had swallowed it, and it was admitted by all who saw it that if it had not, it could not have been swallowed without as much difficulty as was experienced in bringing it up.

I have already alluded to a little adventure we had on the road to Oporto, when we marched all night to surprise the French at Albergaria Nova. This we did, and I got some of the breakfast which was preparing for them; but we did not on the whole distinguish ourselves much after daylight. The wounded at this place, and at Grijon, passed under my observation, but gave nothing of importance; and I was not so fortunate as to gain much at the taking of Oporto, in professional information. We rather, if any thing, retrograded. The French had collected all the boats on their, the northern side of the river, and apparently considered them so secure, as not to think it necessary to place a sufficient guard over them. The consequence was, that soon after the British troops reached the southern bank of the Douro in Villa Nova, the suburb opposite Oporto, one boat was loosened and brought over. The soldiers immediately embarked, crossed, and brought back others, amidst the shouts and vivas of the natives. Sir J. Sherbrooke soon followed, with the whole 29th regiment; and the Portuguese boatmen having procured more boats, ferried me over with my horse. The alarm was perfect. The French, who appear not to have suspected such an accident, fled, leaving horses, mules, and baggage in all directions; every one took to his horse or his heels, and no one thought he could get Oporto fast enough.

The inhabitants seemed afraid to touch any thing themselves, but called out to us to seize every horse and baggage-mule we saw as French. Being the only officer on horseback, I could ride about and take my choice of lots of loaded horses and mules, but I was much too proud to take possession of three or four mules with their baggage. It was not yet considered officer-like to deal in baggage, and so I occupied my time looking for some riding horses, until I lost the British, and was overtaken by Sir J. Milley Doyle at the head of the 16th Portuguese, looking for the English. I offered to shew him the way, as they were only a little before us, and placed myself by his side at the head of his regiment. On turning a corner, I shewed him the 29th Grenadiers, drawn up in line on the rising ground at the end of the road. They as soon perceived us, and after a minute or two, I saw Sir J. Sherbrooke himself face the Grenadier company towards us, and to my astonishment, they very quietly made ready as if on parade. Sir John and the Portuguese called out it was all over with them, and I thought so myself, for, knowing the old grenadiers very well, I took it for granted we were as good as dead. We were too far off to be heard in time, yet close enough to be shot, and it was quite plain they took us for French. I bethought me I had a red round jacket on under my blue undress coat, and as little time was to be lost, I stood up in my stirrups, and opened the blue coat as wide as possible, that none of the red one should be lost. The Grenadiers at this moment came to the present; I thought we were gone; when in an instant I saw them irregularly changing to the recover; they knew me, and had called out the doctor and the Portuguese. I never was so delighted in my life; and galloped up to them forthwith. Sir J. Sherbrooke saluted me with, By God, Sir, if you had not shewn that red jacket, I would have sent you all in a second more to the devil. I knew Sir John very well, and hoped at all events he would have let us gone elsewhere, but he would not hear of it. No, Sir, said he, I would have sent you all to the devil; you should have gone there and no where else: and as it was well known that Sir John would always do what he said, as far as depended on him, there was nothing to be done, but submit. From that day the Portuguese never went into action that I saw without a white band round the left arm. Shortly after this I accompanied the light troops to the front, and had a little skirmish with the French runaways, who were making their escape from the end of every street. Some of them brought out a gun, but on seeing us, and that the road was occupied, as it turned in front of us, they dismounted, and left the gun and the four mules that drew it. This I went to the left and seized, but what to do with a gun and four mules I did not know, more particularly after my failure in horse-stealing; so I settled the matter by taking possession of the best mule, which I carried off, and it served me very faithfully through the Talavera campaign.

Sir John Sherbrooke was a good-hearted man, although rather irascible, and was always willing to do a kind action. If the severity of physic and surgery would admit of a little descriptive episode, I would give you the appearance of the convents of Alisbaça and Batalha when first we saw them. It is better however to refer you to Mr. Beckford's book on Portugal, which is quite as characteristic as it is beautiful. He must have been a gastronome by his unbounded admiration of the kitchen at Alcobaça. It was however a good one, and the live fish did swim about in troughs, placed from one end to the other on the middle of the table, through which the river water constantly flowed; and the gardens were beautiful in the extreme. The padre guardien and the monks were always hospitable. They were obliged in those days by their charter to give a dinner and lodging to every traveller who passed and asked it, and about three half-pence on parting in the morning, and they received us with open arms on all occasions. The dinner was most joyous, the monarch and people of the respective nations were drunk with enthusiasm. I think I now see the jolly old fellows answering to our three times three with a thousand vivas; but they were not all old, and the young ones liked wine. On one occasion, when Sir J. Sherbrooke dined with them, one of the younger ones was also successful in making love; for the English ladies who accompanied the soldiers were not fastidious, and one of them could not resist the solicitations of the handsome monk. He was caught by the soldiers in a situation unhappily for him *pas douteuse.* They immediately placed him on one shutter and the lady on another, and marched them joyously round the cloisters, to the great amusement of the populace assembled at the convent to see the British. The next morning he had disappeared; his trial and punishment were summary; he had been sentenced to a slow death on bread and water, in a small stone cell, from which he was never to he withdrawn alive. The entreaties of Sir J. Sherbrooke prevailed not. The superior honestly admitted, that he would have forgiven the offence at the pressing entreaty of the English general, if it had not been so publicly manifested; but that the character of the order was at stake, and it must be as publicly known that the punishment had been exemplary. Sir John kept up a correspondence with the padre, and after the battle of Talavera, the old gentleman wrote to congratulate him, in more than the usual complimentary terms of the nation. He assured him that he and his order, as well as all Portugal, owed every thing to his gallantry, and that they must ever bless the day that brought him to Alcobaça. Sir John Sherbrooke, with the characteristic feeling of an English gentleman, took him at his word, and wrote him back that he placed such implicit reliance on all he had said as to his feelings towards him, that he could not refrain in reply from asking him for the pardon of the young offending monk as the only, and, at the same time, the greatest favor he could do him. Sir John Sherbrooke, when he told me this story, declared he had felt that day to be one of the happiest of his life, when he received a letter from the superior, enclosing one from the young man, thanking him for his life, and stating the horrible imprisonment he had undergone, and the utter destitution of hope in which he lay, when Sir John's interference and his pardon were announced to him.

### BREACH OF ETIQUETTE IN TAKING FEES FROM MEDICAL MEN, BY DR. J. JOHNSON.

The following Letters, from many others of similar tendency, are submitted to the public, as a set-off against the assertion of one solitary female.

### No. 1.—Dr. DAVIES.

My dear Sir,—Understanding that an attack has been made upon you in the pages of the Lancet by a physician at Cheltenham, for taking fees from medical men who have asked your advice, I beg leave to declare that I have had the pleasure of knowing you about seventeen or eighteen years, and that for the last eight or ten I have been in the habit of coming to London once or twice a year for the purpose of taking your advice in a most distressing and insiduous complaint. You have always received me with kindness, and by the most judicious advice and prescriptions have succeeded in warding off or lessening the attacks of the disease, and since I resigned the public situation I held at Chatham, and retired to the neighbourhood of London, you have attended me at my own dwelling as if I had been a fee-giving patient, and have bestowed much valuable time and attention upon my case. All this you have done, I declare on my honour, without any fee or reward, or without any expectation of getting any, as far as I know. I further declare that I have sent you this letter as soon after I heard of the circumstance as possible, and without having had any communication with you whatever; and I beg of you to make whatever use of it you think proper in making the contents known to the world.

In my own name and for my wife and family I beg you to accept our grateful acknowledgments for your kindness and attention, and to believe that I remain with the most perfect esteem and gratitude, your most obliged and obedient servant,

W. A. DAVIES, M.D.

Late Surgeon to the Hon. East India Company's Depôt at Chatham.

7, Pelham Crescent, Fulham Road.
Wednesday, Nov. 15th, 1837.
To Dr. James Johnson, Suffolk Place.

### No. 2.—Dr. WILSON.

Dear Sir,—I have been much surprised by the assertions made in the letters of Dr. Dickson, which appeared in the Lancet relative to your accepting fees from your professional brethren.

As this accusation must be one calculated to give you much annoyance, I consider myself, from the common feelings of obligation, called upon to acknowledge that I could not have experienced greater attention than was paid to me by yourself during the time I had the Asiatic cholera, four years ago, at which time you found me from home, and a perfect stranger to you.

My friends can testify that on your part neither "fees" nor remuneration in any shape were expected or received.

I have the honour to be, dear Sir, your obliged and obedient servant,

JAMES WILSON.

37, Sackville Street, Piccadilly,

Feb. 10th, 1838.

### No. 3.—Mr. CHURCHILL.

16, Princes Street, Soho—29th Jan, 1838.

Dear Sir,—Your professional liberality having been made the topic of much public controversy, I feel called upon to offer my humble testimony by a reference to the following facts.

Having the honour to be intimately associated with the medical profession, my position informs me of much that is connected with medical men and medical news, and I have heard in such numerous instances medical men refer to professional assistance rendered by you in personal illness, that I had long looked upon you as the "medical man's physician."

But your professional liberality is *not* confined to the circle of your brethren, it takes a wider range. When suffering under indisposition, you saw me in consultation three times in fourteen hours, refusing to take a fee; and on the subsequent illness of my wife, you were constant in your attendance through a protracted illness, and when offered your fee, I well remember your reply:—" O no, we must consider you a chip of the block; I shall be happy to attend you or your family at any time."

I could refer to numerous names in proof of my first statement, and can add that of a non medical friend, to whom your advice has always been gratuitously available, simply from her literary reputation.

I have the honour to be, dear Sir, your faithful and obliged servant,

Dr. Johnson. JOHN CHURCHILL.

No. 4.—Mr. BISHOP.

Maidenhead, Nov. 13th.

My dear Sir,—How fallacious, and, I may add, dishonorable (because false), are the idle attacks made on you respecting your taking fees of medical men. I can bear testimony to the contrary with respect to myself; and on all occasions of my consulting you, either at your own house, or when visiting me at an hotel, have fees been altogether rejected; and moreover, whenever I have sent you a patient, to whom the fee was an object, you have uniformly and generously refused it.

Detraction carries with it its own punishment by reverberation on the author, and the inevitable exhibition of the virtue it attempts to repudiate.

I remain, dear Sir, your's very truly,

J. G. BISHOP.

No. 5.—Mr. DELPH.

Dear Sir,—I was much surprised to see in the Lancet of last week a most unwarrantable attack upon you respecting your manner of receiving fees. As a medical man who have known you for the last 20 years, I can safely affirm (upon oath if required), that as far as regards myself, my family, or any domestic concerning whom I may have consulted you, that you never *would receive* a *fee* in any shape, though often proffered.

I feel much pleasure in thus bearing testimony to the remark, which is almost in every one's mouth, "that Dr. Johnson will never be *rich by the exaction of fees from his patients*," and think that Dr. Dickson, or any other man who brings so open a charge against a public character like yourself, should have substantial grounds to form his charges upon before he commits them to the eye of the public.

I am, dear Sir, very faithfully your's,

N. DELPH.

To Dr. J. Johnson,
11, Alfred Place, St. George's Southwark.
29th Sept. 1837.

No. 6.—Mr. SIMPSON.

Hammersmith, Sept. 27th, 1837.

My dear Sir,—I perceive a Dr. Dickson has made a most disgusting personal attack upon you in the last Lancet, accusing you of demanding fees from medical men. Now it will ever be in my remembrance, that after being in a court of justice several hours as a witness in the trial of myself and Dr. Collier, you, although a perfect stranger to me, returned the guinea you received with the subpœna; and you were the only physician that did so. Another physician, whom I had been acquainted with, and called in, asked Mr. Wakley what he should do with the guinea, who replied, " why give it to the losing party to be sure." I once sent you a patient, whom I told you could only afford half-a-fee, and you took nothing from him. With this experience of your character, I feel almost inclined to take up the cudgels. God help reviewers—for authors are of the irritabile genus. But this man's attack is contemptible.

I am, dear Sir, your's sincerely,

W. SIMPSON.

No. 7.—Mr. LISTON.

13, Old Burlington Street,
Jan. 23d, 1838.

My dear Sir—I can bear testimony to your kindness to one medical man, Mr. Craig, from Paisley, and to your peremptory refusal of remuneration, when personally pressed upon you. You on that occasion stated that you never had taken, and never would take, a fee from any one in the profession.

Believe me your's faithfully,

Dr. James Johnson. W. J. LISTON.

---

No. 8.—Mr. EDMUNDS.

Kineton, Dec. 7th, 1837.

Dear Sir,—Respecting the charge made against you by Dr. Dickson, as published in the Lancet, I am in possession of a letter with which you favoured me in the year 1826, the postscript to which I annex. In the Autumn of that year I suffered very severely, and for many weeks, with cardialgia, to relieve which I tried various means, but without avail. I then wrote you a statement of my case, which had your immediate attention, and I received a most kind and gentlemanly reply, pointing out a plan of treatment from which I derived almost immediate, and I am happy to say, permanent benefit. I believe you have alluded to my case in your work on Morbid Sensibility of the Stomach. Having read the correspondence betwixt you and Dr. Dickson in the Lancet, I wrote to the Editor about three weeks since, stating that I was indebted to you for your advice, and copied the postscript of your letter, requesting the favour of its insertion in the Lancet, but as it has not yet appeared in that publication, I am induced to trouble you with this. Again I beg to thank you for your very kind and considerate attention to my case, for which I feel the more indebted being personally unknown to you. Allow me to subscribe myself,

My dear Sir, your's truly obliged,

J. EDMUNDS.

*Postscript to your Letter.*

P.S. I never took a fee from a brother practitioner in my life, and shall not now begin. Do not hesitate to command my advice whenever you think fit.

Dated 13th Dec. 1826.

---

No. 9.—Dr. CHRISTIE.

Dear Sir,—In reference to the ill-advised accusation of Dr. Dickson against you, I have to beg you to accept of my apologies for not having earlier tendered my humble testimony in favor of the *very universally acknowledged liberality of your practice in your profession*, and which I feel I certainly ought to have done before.

If Dr. D. will take the trouble of calling on me, I shall have much pleasure in *attempting* to express to him something like the feeling of gratitude I shall ever entertain towards you for your ready, unremitting, and most solicitous attendance upon, and attention to different branches of my family during periods of illness, and for the very valuable services you have PERFECTLY GRATUITOUSLY rendered to them.

Admirably liberal as the medical practitioners of this country undoubtedly are in affording every possible assistance in their power *gratuitously* one to another in case of illness, if any member of the profession in London were asked to name the practitioner who in this great metropolis was the *least mercenary* in the practice of his profession, the *most ready* to render *gratuitous* assistance to his professional brethren or to their families, and the *most liberal* towards non-professional persons, I firmly believe, if it were practicable to make the selection, Dr. JOHNSON, of Suffolk Place, would be the gentleman fixed upon.

I am persuaded there is no practitioner here who is not thoroughly assured that you are liberal in your practice almost—if not altogether—to a failing, (though much to your honor and credit) even to those who are wholly unconnected with the profession, whenever you ascertain that such persons are at all in situations to require charitable aid.

Under such circumstances, so perfectly certain might you have been that Dr. D.'s charge against you of illiberality towards your professional brethren *must* have carried with it *its own utter refutation* to the minds and knowledge not only of every medical man who has the pleasure of knowing you, but almost of every one who ever heard of your name, that you will forgive my saying, I should have thought you never would have descended to offer a denial of the accusation.

Allow me to entreat now that you will accept of an expression of the *deepest* sense of gratitude on my part towards you for your very able and *wholly gratuitous* services to my family, and believe me to remain, with great respect,

Dear Sir, very truly,

— CHRISTIE, M.D., et M.R.C.S.L.

37, Great James Street, Bedford Row,
22nd Jan. 1838.
To Dr. Johnson.

---

No. 10.—Dr. CLENDINNING.

My dear Sir,—I perceive in a letter of your's in the last Lancet a list of medical practitioners, including myself, as having been attended by you without remuneration, and considering myself in some sort summoned, by the publication of my name, to state the truth, the whole truth, and nothing but the truth, so far as known to me, touching your manner of dealing with such of your medical brethren as ask your advice for themselves or members of their families, I have pleasure in acknowledging that I for one am indebted to you for repeated services of that kind. You have, on several occasions, given myself personally the advantage of your valuable advice and able assistance, and always gratuitously.

You also attended, now some seven years since, a married lady, a near relation of mine, during a tedious illness, which occurred while your patient was on a visit in my house, and for that attendance, unless I am strangely deceived, you declined all remuneration on the ground that the lady was, for the time at least, a member of my family. From those facts known to myself personally, and my general knowledge of your character, I conclude that your attendance on professional men and their immediate families has always been given on the usual liberal terms, and have no doubt that that conclusion will be confirmed by such of the practitioners named in your letter as may feel themselves, as I do, called on, and answer your appeal.

Believe me, my dear Sir, your sincerely obliged,
JOHN CLENDINNING.

16, Wimpole Street,
Jan. 29, 1838.

---

Dr. Johnson appeals to his brethren whether it is probable that he would break through a principle on which he has acted for thirty years and more, for the sake of getting two or three guineas from the wife of Mr. Gibbon. Since the charge that has been brought against him, he has learnt, not only from Mrs. Gibbon, but from the physician who attended Mr. Gibbon on his death-bed, that the regular fees were paid to the physician in question by the executors, and received for the following reasons, viz.—that Mr. Gibbon had retired, with competence, from the service and the profession—and that he and others in similar circumstances, did not consider themselves justifiable in absorbing the time and labours, *gratuitously*, of men supporting their families by the arduous practice of their profession. Such individuals prefer giving the ordinary remuneration to their attendants, rather than lie under an irksome obligation, or be obliged to make costly presents to the wives or daughters of their medical prescribers. Such line of argument is unanswerable, although Dr. Johnson can most solemnly and conscientiously aver that he never once acted upon it, or received a fee, directly or indirectly, from a brother officer of the profession. He has now done with the subject.

---

### NEGATIVE VIRTUES: OR, A NEW METHOD OF RISING ON THE RUIN OF OUR NEIGHBOURS.

WE call the attention of all young candidates for Hospitals, Infirmaries, or Dispensaries, to the following *original*, and truly liberal address, published in the "CHELTENHAM JOURNAL," for Monday, March the 5th, 1838.

"CHELTENHAM DISPENSARY.

*To the Governors and Subscribers to the Cheltenham Dispensary and Casualty Hospital.*

"Ladies and Gentlemen,—I know not what reason your Secretary will give for having *omitted* to advertise you that there have been for sometime back two Vacancies in the Office of Physician to your Institution, the one arising out of the retirement of Dr. Conolly to Charlton, the other occasioned by the departure of Dr. Bernard to the West Indies. I beg to present myself as a Candidate for one of these Appointments; but, instead of following the beaten course of detailing my good intentions,—should I succeed in obtaining your suffrages and support,—I shall tell you what I will NOT do:—

"I will not accept the honour of the name without myself doing the duties of Physician.

"I will in no case shew a lack of knowledge of the better resources of my art, by abstracting the life-blood of the poor—whose diseases, for the most part, spring from defective food and vitiated air.

"I will not see the Patients maltreated, mutilated, or MURDERED, without raising my voice against the authors of such barbarity.

"I will not consent to witness cruel operations, however dazzling, or calculated to answer the ends of *rising operators*, which may be prevented by judicious *internal* treatment.

"I will not allow professional or other etiquette to interfere with the welfare of the Patients entrusted to my care.

"I will not convert the medicines of the Charity to the use of my private patients.

"I will not sanction false Returns of the medicines used, or of the result of the general treatment or numbers of the Patients who have applied for assistance.

"I will not interfere with the duties of the Chaplain to the Institution, by discussing religious subjects, or praying *with* the sick, however much in my heart I may pray FOR them.

"I will not retain office after the expiration of one year from the date of my election, if I shall fail satisfactorily to demonstrate to the Subscribers that during that time I have increased the number of applicants for relief, and added to the usefulness and respectability of the Charity.

"I have only in conclusion to say, that I shall shortly submit to your attention Testimonials, from eminent individuals of the Army Hospital and General Staff, which will shew that, while in charge of extensive Military Hospitals, I *did not neglect* the duties intrusted to me. The evidence I shall adduce upon this head shall be such as the ingenuity of the base may by no possibility neutralize.

"I remain, with much respect,
"Ladies and Gentlemen,
"Your most obedient humble servant,
"S. DICKSON, M.D."

"15, Imperial Square, Cheltenham,

"March 3, 1838."

Well may Dr. Dickson say that he does not mean to follow "*the beaten course*" in these canvasses for public institutions! Most candidates address the governors by regretting the lamented death or resignation of their predecessors, &c. &c. Not so Dr. Dickson! He indirectly, but quite *unequivocally* accuses the Medical Officers of the Cheltenham Dispensary, of ignorance, negligence, peculation, robbery, mutilation—MURDER (!!) of the unfortunate sick who come under their care!! What must the public at large think of the Medical Profession, when a physician, boasting the special patronage of the heads of the army medical department, can issue an address—so atrocious a libel on his brethren, and which, we will aver, has never been paralleled in any age or any nation! The author of it is evidently more ignorant of law than he is of physic. He thinks that *insinuations* are not libellous. If Dr. Connolly or Dr. Bernard, whose names are adduced in the paper, choose to prosecute, they are as sure of a verdict as they are of their own existence. No jury, between the Land's End and Caithness, would hesitate, one instant, to pronounce the address a foul and calumnious libel, and award heavy damages.

Will the Governors and Subscribers of the Cheltenham Dispensary permit these foul aspersions to be thrown on their Medical Officers of the Institution, without meeting to repel them? We think they cannot sit quiescent under such insinuations. Dr. Dickson has now thrown off the mask, and undertaken the task of rendering the whole profession disgraceful in the eyes of the community at large! We wish him joy of his crusade.

---

### ROYAL COLLEGE OF SURGEONS IN IRELAND.

*Bye-laws relating to Education, and to the Examination of Candidates for Letters Testimonial.*

### HALF-YEARLY EXAMINATIONS.

The President, with the Vice-President, and the Members of the Courts of Censors and Assistants, or a majority of them assembled together, shall appoint, by a majority of voices, from among themselves, four or more Members, with the President, or Vice-President, to examine the registered pupils as to their proficiency in their studies every half-year, in the months of April and November, of which examinations due notice shall be given by summons, and by advertisement in the newspapers.

The pupils shall be divided into two classes, and each class shall be examined in the presence of the Members and Licentiates of the College, for such length of time, not being less than one hour, as the Examiners may think proper, and the name of every pupil who shall have answered such examination to the satisfaction of the said Examiners, shall be enrolled by the Secretary in a book provided for that purpose, and the same shall be certified by the President to the Court of Censors, otherwise not.

The first or junior class shall be examined as to their knowledge of the elements of anatomy and physiology, the descriptive anatomy of the bones, muscles and joints, and of the cavities of the thorax and abdomen.

The second or senior class, shall be examined on the functions of digestion, absorption, circulation and respiration, the anatomy of the vascular and nervous system, and of the organs of sense.

Candidates shall be liable to be examined respecting their knowledge of any of the preceding subjects, or of any other subject not here enumerated, at their final examination for Letters Testimonial, as heretofore.

### EXAMINATION IN PHARMACY.

A Court of Examiners, consisting of seven members, shall be elected by ballot on the first Monday in February, in each year, to examine the registered pupils in chemistry, materia medica, and pharmacy. This Court shall elect from amongst themselves a chairman and deputy chairman; which chairman, or in his absence, the deputy chairman, with any four or more of such Court, shall be authorized and required to hold such examination.

This Court shall examine every registered pupil who shall require it, and who shall have been engaged in the study of his profession for three years at least, and shall have attended two courses of lectures on Chemistry, or one course of lectures on general Chemistry, and one course on the practical details of Chemistry, one course of lectures on Materia Medica and Pharmacy, and one course of lectures on Medical Jurisprudence.

Such registered pupils shall be publicly examined on the general principles and practical details of chemistry; on materia medica and pharmacy, including the composition, qualities, and uses of remedies, and the botanical characters of medical plants, with the method of compounding and administering medicine; and also upon toxicology and medical jurisprudence: and if approved of and found qualified to compound and administer drugs and medicines, the Court shall certify the same to the President and Court of Censors; and if that Court thereafter shall grant to such pupil the Letters Testimonial of the College, such certificate of his qualification in pharmacy shall be delivered to him along with such Letters Testimonial.

### Examination in Midwifery.

A Court of Examiners, consisting of seven members, shall be elected by ballot on the first Monday in January, in each year, to examine such members, or licentiates, as become candidates for the license, diploma, or certificate to practise midwifery. This Court shall elect from amongst themselves a chairman and deputy chairman, which chairman, or in his absence, the deputy chairman, with four or more members of such Court, shall be authorized and required to hold such examination.

Candidates for the midwifery diploma, license, or certificate, shall have attended one course of lectures on Midwifery and Diseases of Women and Children, delivered by a Professor or Lecturer in some school of medicine or surgery recognized by the Court of Censors; he shall also have attended the practice of a lying-in hospital, recognized by the Midwifery Court, for a period of six months; or, the practice of a dispensary for lying-in women and children, recognized by the Midwifery Court, and devoted to this branch of surgery alone; and also shall have conducted thirty labour cases at least.

Candidates for the midwifery diploma, license, or certificate, shall be publicly examined on the structure and functions of the organization of the female, the growth and peculiarities of the fœtus, the practice of midwifery, and the diseases of women and children; and if approved of, shall receive a license or diploma under the hands of the Examiners, certifying the same, to which the College seal shall be attached. Should a candidate be rejected, he shall not be again admitted to an examination until a period of three months shall have elapsed; and he shall then be obliged to produce satisfactory evidence of his having been engaged in the study of this branch of surgery subsequent to such rejection.

No Member or Licentiate of the College is, or shall be, authorized or licensed to practise midwifery, or to give instructions therein, or to grant certificates of attendance on lectures thereon, unless he shall have been examined by the Midwifery Court of Examiners, and shall have received the diploma or license of the College to practise that branch of surgery; or unless he shall have been recognized as a midwifery practitioner previous to the 1st of May, 1831.

The Court shall also be authorized and directed to examine in the same manner, and subject to the same regulations, every registered pupil who shall require it, and who shall have been engaged in the study of his profession for the period of three years, and shall have laid before the Court the documents and certificates required to be produced by candidates for the midwifery diploma; and if they consider that such registered pupil has pursued his studies in this department diligently and effectually, and that they believe, from his answering, that he has acquired a sufficient knowledge of this branch of surgery, they shall certify the same, under the hand of the chairman, to the President and Court of Censors, and if at any time thereafter he shall have the Letters Testimonial of the College granted to him, they shall also grant to him the usual midwifery diploma, upon his passing such examination as they shall deem necessary.

### Examination for Letters Testimonial.

Every registered pupil or apprentice shall be admitted to an examination for Letters Testimonial, if he shall have proved and showed that his professional education has been in all respects conformable and agreeable to the provisions and enactments of the bye-laws and rules of the College, and shall have laid before the Court the following documents:—

1. A receipt showing that he has lodged a sum of thirty guineas in the Bank of Ireland, to the credit of the President and for the use of the College.

2. A certificate signed by the President or Vice-President and two of the Court of Censors, that he has passed an examination as to his acquaintance with the Greek and Latin classics in the following books: the works of Sallust, the first six books of the Æneid of Virgil, the Satires and Epistles of Horace, the Greek Testament, the Dialogues of Lucian selected by Walker, and the first four books of Homer's Iliad; or a certificate that he has entered as a student into Trinity College, or into any British University granting degrees in the arts, where he has already undergone a classical examination.

3. If he be an apprentice, his indenture of apprenticeship regularly registered, with a certificate signed by the Member or Licentiate to whom he has been indented, that he has fully and perfectly served such apprenticeship for the full term of five years.

4. If he be not an apprentice, certificates showing that he has been engaged in the study of his profession in some hospital or school of medicine or surgery for five years, and that he has attended lectures or hospitals for three winter seasons, at least, in Dublin, Glasgow, London, or Edinburgh.

5. A certificate, signed by the President or Vice-President, that he has passed the two half-yearly examinations required by the bye-laws.

6. A certificate, signed by the chairman or deputy chairman of the Pharmacy Court, that he has passed the examination in chemistry, materia medica, and pharmacy.

7. A certificate, signed by the chairman or deputy chairman of the Midwifery Court, that he has passed the examination in midwifery and on the diseases of women and children.

8. Certificates of attendance on a surgical hospital containing fifty patients, for a period of three years at least.

9. Certificates of attendance on three courses of lectures on Anatomy and Physiology, three courses of lectures on the Theory and Practice of Surgery, and of the performance of three courses of Dissections, accompanied by Demonstrations: also certificates of attendance on two courses of lectures on Chemistry, or one course of lectures on General, and one on Practical, Chemistry; one course of lectures on Materia Medica; one course of lectures on the Practice of Medicine; one course of lectures on Midwifery; and one course of lectures on Medical Jurisprudence.

10. A thesis, essay, or dissertation in Latin or English, on any of the following subjects: Anatomy, Physiology, Surgery, the Practice of Medicine, Chemistry, Materia Medica, Midwifery, or Medical Jurisprudence; or in place of such dissertation, a series of cases collected in the hospital in which the candidate has attended, illustrated by comments or observations.*

### LIQUOR LYTTÆ.

The Acetum Cantharidis of the Pharmacopœia, is a comparatively inert preparation. The LIQUOR LYTTÆ, as prepared by Mr. Snowdon, in the Haymarket, is one of the most elegant and useful rubefacients, counter-irritants, or vesicatories, which we have ever employed. A single painting or varnishing of the part, by means of a camel's-hair pencil, produces a rubefacient effect, more lasting than a mustard-poultice—a couple of applications with two or three minutes' interval, will counter-irritate effectually—and three applications, with similar intervals, will, in a great majority of cases, vesicate the part in five or six hours. A piece of linen wetted with the LIQUOR LYTTÆ, and laid on the skin for five or six minutes, is sure to blister, in little more than half the time required for a common blister-plaister. The advantages of the Liquor Lyttæ are numerous. To angular parts, as about the neck, it is difficult to apply or keep on a blister. With children, also, there is much trouble in keeping the emp. lyttæ on the parts. The *application* of the *Liquor Lyttæ* gives no pain, and its subsequent blistering effects cannot be resisted by the impatient patient. The odour is the same as that of the College preparation, but the strength is infinitely greater.

### SUBSTITUTE FOR CINCHONA.

By a Letter which we have received from Mr. MEASE of Philadelphia, we learn that the General or President of Peru and Bolivia has prohibited the exportation of Cinchona from that country for five years to come. Europeans, therefore, will be obliged to search about for a substitute. Mr. Mease has sent us a packet of the EUPATORIUM PERFOLIATUM, more commonly known by the trivial name of "BONE-SET," whose tonic powers, he observes, have been tested for half-a-century, and whose efficacy in the cure of intermittents is acknowledged. The specimen may be examined in Suffolk Place, by any gentleman desirous of seeing it. We have also sent a part of it, with Mr. Mease's letter, to the Medico-Botanical Society.

*** Mr. Mease's paper, on the Stings of Venomous Insects, in our next.

* For the remaining elucidations and explanations of these laws and regulations, we have not room in our Journal, being much crowded and overcharged this quarter.—J. J.

